# Recent Advances of MicroRNAs, Long Non-coding RNAs, and Circular RNAs in Preeclampsia

**DOI:** 10.3389/fphys.2021.659638

**Published:** 2021-04-30

**Authors:** Ailing Chen, Renqiang Yu, Shiwen Jiang, Yankai Xia, Ying Chen

**Affiliations:** ^1^Translational Medicine Laboratory, Research Institute for Reproductive Health and Genetic Diseases, The Affiliated Wuxi Maternity and Child Health Care Hospital of Nanjing Medical University, Wuxi, China; ^2^Department of Neonatology, The Affiliated Wuxi Maternity and Child Health Care Hospital of Nanjing Medical University, Wuxi, China; ^3^Research Institute for Reproductive Health and Genetic Diseases, The Affiliated Wuxi Maternity and Child Health Care Hospital of Nanjing Medical University, Wuxi, China; ^4^State Key Laboratory of Reproductive Medicine, Institute of Toxicology, Nanjing Medical University, Nanjing, China

**Keywords:** preeclampsia, microRNAs, long non-coding RNAs, circular RNAs, trophoblast, biological function

## Abstract

Preeclampsia is a clinical syndrome characterized by multiple-organ dysfunction, such as maternal hypertension and proteinuria, after 20 weeks of gestation. It is a common cause of fetal growth restriction, fetal malformation, and maternal death. At present, termination of pregnancy is the only way to prevent the development of the disease. Non-coding RNAs, including microRNAs, long non-coding RNAs, and circular RNAs, are involved in important pathological and physiological functions in life cycle activities including ontogeny, reproduction, apoptosis, and cell reprogramming, and are closely associated with human diseases. Accumulating evidence suggests that non-coding RNAs are involved in the pathogenesis of preeclampsia through regulation of various physiological functions. In this review, we discuss the current evidence of the pathogenesis of preeclampsia, introduce the types and biological functions of non-coding RNA, and summarize the roles of non-coding RNA in the pathophysiological development of preeclampsia from the perspectives of oxidative stress, hypoxia, angiogenesis, decidualization, trophoblast invasion and proliferation, immune regulation, and inflammation. Finally, we briefly discuss the potential clinical application and future prospects of non-coding RNA as a biomarker for the diagnosis of preeclampsia.

## Introduction

Preeclampsia (PE) clinically manifests as new hypertension, proteinuria, or other clinical symptoms and signs of impaired end-organ function after 20 weeks of pregnancy (Duhig et al., 2018), and has an incidence of about 5–8% (Souza et al., 2013). PE is divided into early-onset PE and late-onset PE by 34 weeks. Early-onset PE is widely acknowledged to have defective placentation in the early stages of pregnancy, while late-onset PE appears to be driven by oxidative changes in the placenta induced by a mismatch between normal maternal perfusion and fetoplacental demands, coupled with a maternal genetic predisposition to cardiovascular and metabolic disease (Burton et al., 2019) and/or a high BMI (McLaughlin et al., 2018). Compared with late-onset PE, early-onset PE usually has more severe complications (Lisonkova et al., 2014) and smaller placental perfusion fraction (Sohlberg et al., 2014). The pathophysiological characteristics of early-onset PE are due to shallow trophoblast invasion, which affects the remodeling of spiral arteries, resulting in decreased uterine placental blood flow and oxygenation, leading to increased placental oxidative stress. Late-onset PE is manifested as a result of villous overcrowding at term. Due to limited gas exchange and nutrient supply, cellular growth is competitively inhibited, which ultimately leads to placental oxidative stress (Salomon et al., 2017). Abnormal remodeling of the uterine spiral artery and damage to the systemic vascular endothelial system are key to the onset of PE. Insufficient reconstruction of the uterine spiral artery may be related to the following factors. First, in the maternal inflammatory response, a significant increase in the level of hypoxia-inducible factor (HIF) protein in the placenta of patients with PE indicates that hypoxia is a major risk factor for PE (Ali et al., 2019). Decreased placental perfusion and oxidative stress are key to stimulating the release of cytokines, anti-angiogenic factors, and related downstream products. Eventually, inflammation and endothelial dysfunction of numerous organs and systems (including the vasculature, kidney, liver, and brain) have been observed in PE, especially early-onset PE (Kim et al., 2017). Second, vascular endothelial dysfunction is a possible factor, as the expression levels of soluble fms-like tyrosine kinase 1 (sFlt-1) and soluble endoglin (sEng) are elevated in the circulating blood and placenta tissue of patients with PE (Steegers et al., 2010). sFlt-1 and sEng are anti-angiogenic proteins that antagonize pro-angiogenic factors, including vascular endothelial growth factor (VEGF) and placental growth factor (PLGF), leading to placental vascular dysplasia (Steegers et al., 2010). Third, specific human leukocyte antigens on trophoblast cells in blood vessels may interact with natural killer cells and T cells of the maternal immune system, thereby altering the normal maternal–fetal immune tolerance (Wang X.Q. et al., 2018).

Non-coding RNA (ncRNA) can be transcribed from the genome but does not produce a functional protein, and can be divided into housekeeping and regulatory types. Housekeeping ncRNA includes ribosomal RNA, transfer RNA, and small nucleolar RNA, whereas regulatory ncRNA generally includes microRNA (miRNA), long non-coding RNA (lncRNA), and circular RNA (circRNA) (Gomes et al., 2013). lncRNA and circRNA can share the miRNA response elements of linear messenger RNA (mRNA). They competitively bind to miRNA, thereby affecting the regulatory function and gene expression of miRNA (Lukiw, 2013). The competitive endogenous RNA (ceRNA) hypothesis indicates that an interaction network exists among miRNA, lncRNA, circRNA, and mRNA. ncRNA is not a so-called junk product but rather plays important biological roles in cell physiology, development, and metabolism. It can regulate transcription and post-transcriptional gene expression levels in various diseases (Yoon et al., 2013). Whether all transcripts are functional remains controversial, but it is clear that a large number of ncRNAs have functions. These functional ncRNAs are of great significance for the maintenance of normal cell functions as well as the occurrence and development of abnormal biological functions, and can regulate epigenetic inheritance, transcription, and post-transcriptional biological processes, in addition to participating in the regulation of multiple signaling pathways underlying cellular processes.

Whole-genome analysis of placenta-derived miRNA, lncRNA, and circRNA led to the identification of abnormally expressed ncRNAs associated with PE. Studies have confirmed that ncRNAs play important roles in regulating the development and function of the placenta and the pathogenesis of PE. The abnormal placental transcriptome of PE is affected by both genetic and epigenetic pathways. However, how differentially expressed genes and transcripts regulate the occurrence and development of PE has not been fully elucidated. In this review, we summarize recent advances of research into ncRNAs (including miRNAs, lncRNAs, and circRNAs) in the pathogenesis of PE.

## MiRNAs in PE

### MiRNAs

MicroRNA is single-stranded RNA with a length of 22–28 nt, and is formed from a miRNA precursor with a hairpin ring structure through the sequential activities of the Drosha and Dicer ribonuclease III endonucleases. miRNA is conserved evolutionarily, and its expression shows tissue and stage specificity. It is an important element in physiological and pathological processes in various cells. miRNA affects the stability of target genes and inhibits the expression of target proteins. Mature miRNA binds to an RNA-induced silencing complex (RISC), and then specifically binds to the target RNA, causing its degradation. In animal cells, most miRNAs are not completely complementary to their target genes. The seed region of miRNA is complementary to the 3′ untranslated region (3′ UTR) of the target gene to prevent transcription (Djuranovic et al., 2011). miRNA is involved in a broad range of life processes including cell proliferation, differentiation, apoptosis, and migration (Sun and Lai, 2013). It has been speculated that miRNA regulates one third of human genes. During pregnancy, miRNA is involved in regulating the initiation of pregnancy and ensuring stability throughout pregnancy through the regulation of inflammation, immune tolerance, angiogenesis, apoptosis, and other processes (Schjenken et al., 2016).

### MiRNAs in the Pathogenesis of PE

Abnormally expressed miRNAs in the placenta participate in the occurrence and development of PE in terms of maternal-fetal hypoxia, angiogenesis, immunity, and inflammation through different cellular pathways and modes of action. Chim et al. (2008) first reported the presence of miRNA in the circulating blood of pregnant women. In a panel of TaqMan microRNA Analyses for 157 well-established miRNAs, they found that 17 occurred at concentrations >10-fold higher in the placentas than in maternal plasma and were undetectable at the end of pregnancy. The expression levels of multiple miRNAs such as miR-517-5p and miR-520a-5p in the circulating blood of patients with PE in early pregnancy are elevated, which consistent with those in the placenta of pregnant women with PE (Hromadnikova et al., 2013). Table 1 shows differential expression of miRNA involved in PE pathophysiology.

**TABLE 1:** miRNAs in the pathogenesis of Preeclampsia.

miRNAs	Source	Expression	Target	Function	References
miR-106b	Placental tissues and peripheral blood mononuclear cells	upregulated	MMP-2	invasion and proliferation	Li et al., 2017c; Eghbal-Fard et al., 2019
miR-126	Placental tissues and endothelial progenitor cells	downregulated	PIK3R2	proliferation, differentiation, migration and vascular sprouting	Yan et al., 2013
miR-128a	Placental tissues	upregulated	Bax	apoptosis	Ding et al., 2016
miR-133	Placental tissues	upregulated	Rho/ROCK signaling pathway	apoptosis	Zhang W.M. et al., 2019
miR-136	MSCs, exosomes of peripheral blood and umbilical cord mesenchymal stem cells	upregulated	BCL2, VEGF	suppress proliferation and promote apoptosis of MSCs	Ji et al., 2017; Motawi et al., 2018
miR-141	Placental tissues, peripheral blood	upregulated	CXCL12β, MMP-2, p62, LC3B, ROCK1, RhoA, EG-VEGF	apoptosis, invasion and vascularization	Wang C.Y. et al., 2019; Wu J.L. et al., 2019
miR-141-3p	HUVECs	upregulated	Notch2	tube formation, migration, invasion and apoptosis	Ling et al., 2021
miR-145-5p	Placental tissues and umbilical cord blood	downregulated	FLT1, Cyr61	trophoblast viability and invasion	Hromadnikova et al., 2017; Wen et al., 2018; Lv et al., 2019; Wang Y. et al., 2019
miR-155	Placental tissues and maternal plasma	upregulated	eNOS	invasion and migration	Li et al., 2014; Gan et al., 2017
miR-16	Placental tissues	upregulated	Notch2	proliferation, migration and invasion	Yuan et al., 2020
miR-17	Placental tissues	upregulated	ephrin-B2/Eph-B4 receptor	angiogenesis	Wang et al., 2012
miR-181a-5p	Placental tissues and maternal plasma	upregulated	IGF2BP2	Invasion, proliferation, apoptosis and migration	Wu et al., 2018; Huang et al., 2019; Gusar et al., 2020
miR-196a-5p	Placental tissues	downregulated	SNX16, BHLHE40	trophoblast viability, migration, and invasion	Zhang L. et al., 2013
miR-203	Placental tissues and maternal vessel	upregulated	VEGFA	proliferation, migration and invasion	Wang Y. et al., 2016; Liu et al., 2018
miR-205	Placental tissues and maternal plasma	upregulated	MED1	cell differentiation	Mouillet et al., 2010; Yang et al., 2015
miR-20a	Placental tissues	upregulated	FOXA1	proliferation, migration and invasion	Wang Y. et al., 2014; Yuan et al., 2020
miR-20b	Placental tissues	upregulated	MMP-2, MCL-1	proliferation, migration and invasion	Jin et al., 2017; Zhang S.S. et al., 2020
miR-210	Placental tissues and maternal plasma	upregulated	HIF-1a, HIF-2α, HSD17B1, E2, PTPN2, KCMF1, THSD7A, NOTCH1	proliferation, migration apoptosis and invasion, vascular remodeling and growth, tube-like formation capabilities	Ishibashi et al., 2012; Zhang et al., 2012; Luo et al., 2014; Seyom et al., 2015; Song et al., 2015; Wu et al., 2015; Luo et al., 2016; Zhao et al., 2016; Li J. et al., 2019; Wang R. et al., 2019
miR-214-5p	Placental tissues	upregulated	Jagged 1/Notch	proliferation, migration and invasion	Gong et al., 2020a
miR-218	Placental tissues	upregulated	LASP1	invasion	Fang et al., 2017
miR-218-5p	Placental tissues	downregulated	TGFB2	invasion and differentiation	Brkic et al., 2018
miR-299	Placental tissues	upregulated	HDAC2	invasion and migration	Gao et al., 2018
miR-29b	Placental tissues and dMSCs	upregulated	MCL1, MMP-2, VEGFA, ITGB1,HDAC4,	apoptosis, invasion, angiogenesis, proliferation, migratory and tubule formation	Li et al., 2013; Xin et al., 2017
miR-30a-3p	Placental tissues	upregulated	IGF-1	invasion and apoptosis	Lu et al., 2020
miR-335-5p	Placental tissues	upregulated	ROS/p53, Sp1	migration and EMT	Jiang et al., 2015; Lu et al., 2020
miR-342-3p	Placental tissues and maternal plasma	upregulated	ID4, PDGFRA	cell viability, apoptosis and invasion, proliferation, migration, and G1/S phase transition	Wu et al., 2012; Yang and Guo, 2019; Han et al., 2020
miR-346	Placental tissues and maternal plasma	upregulated	LRP6	proliferation, migration and invasion	Tsai et al., 2017; Zhang L. et al., 2020
miR-376c	Placental tissues and maternal plasma	downregulated	ALK5, ALK7, TGF-b/Nodal	proliferation, migration and invasion	Fu et al., 2013
miR-378a-5p	Placental tissues	downregulated	Nodal	cell growth, survival, migration and invasion	Luo et al., 2012
miR-454	Placental tissues	downregulated	Nodal/ALK7, EPHB4	proliferation and invasion	Shi et al., 2019
miR-494	dMSCs	upregulated	CDK6, CCND1, VE Wang R. et al., 2019 GF	arrests G1/S transition, migration and impairs HUVEC capillary formation	Chen S. et al., 2015
miR-495	Umbilical cord tissue and dMSCs	upregulated	-	cell cycle, apoptosis, migration, invasion and tube formation	Li et al., 2017b
miR-517	Placental tissues and maternal plasma	upregulated	sFLT1, ERK/MMP-2 pathway	proliferative and invasive	Anton et al., 2015; Fu et al., 2018; Hromadnikova et al., 2019
miR-520g	Maternal serum	upregulated	MMP-2	migration and invasion	Jiang et al., 2017
miR-646	Human peripheral blood-derived endothelial progenitor cells	upregulated	VEGF-A, HIF-1α	differentiation, migration, angiogenesis	Dong X. et al., 2020

#### Oxidative Stress and Hypoxia Damage

During pregnancy, the production of reactive oxygen species (ROS) in the serum of pregnant women increases due to the normal systemic inflammatory response (Yang et al., 2012). Vascular remodeling disorders in PE lead to decreased placental perfusion and oxygenation, intensifying oxidative stress associated with the production of ROS and toxic lipid peroxides (Myatt, 2010). In pregnant women with PE, miR-335-5p is significantly up-regulated. ROS can increase the expression level of miR-335-5p in trophoblasts in a p53-dependent manner, leading to down-regulation of Sp1 and subsequent inhibition of the epithelial-mesenchymal transition (EMT) and cell migration (Lu et al., 2020). Compared with gestational week-matched controls, the circulating level of miR-195 was reduced in pregnant women with PE, and the expression levels of FAD-dependent oxidoreductase domain containing 1 (FOXRED1) and pyruvate dehydrogenase phosphatase regulatory subunit (PDPR) in the placenta were significantly elevated. Under chronic hydrogen peroxide stimulus, miR-195 expression decreased. miR-195 could suppress mitochondrial energy production via targeting of FOXRED1 and PDPR, which led to trophoblast cell apoptosis under oxidative stress (Wang H. et al., 2018). Perfluorooctanesulfonic acid, which induces oxidative stress, significantly increases miR29-b during the pathogenesis of PE (Sonkar et al., 2019).

The initial stage of placenta formation occurs in a relatively hypoxic environment. This environment can promote trophoblast cell invasion and blood vessel formation. However, continuous hypoxia leads to abnormal trophoblast differentiation and insufficient invasion, resulting in uterine spiral artery recasting disorders and decreased placental blood perfusion. Eventually, the angiogenetic function of the placenta becomes abnormal (Berkane et al., 2007). miR-210 is an important hypoxia sensor regulated by Hipper-Lindau tumor suppressor genes, HIF-1α, HIF-2α, and the inducible factor prolyl hydroxylase (Seyom et al., 2015). miR-210 is significantly elevated in PE plasma and placenta, which is associated with cell migration, vascular remodeling, and growth during embryo formation (Zhao et al., 2016). Patients with mild or severe PE have about four or ten times the level of miR-210 in the plasma relative to healthy people, respectively (Wu et al., 2015). Under hypoxic conditions, miR-210 expression in trophoblasts is significantly up-regulated. Histopathological results show that high expression of miR-210 can lead to down-regulation of HSD17B1 and E2 levels (Song et al., 2015), while low expression of miR-210 can lead to down-regulation of PTPN2 in the PDGFR–Akt pathway and thus promote cell proliferation, migration, and invasion (Li J. et al., 2019). 17-β hydroxylase is the receptor of miR-210, and is mainly expressed in placental syncytiotrophoblasts. Its level falls significantly before PE occurrence. Sorting nexin 16 (SNX16) plays a negative regulatory role in cell migration and tumorigenesis. The expression of miR-196a-5p is reduced in the PE placenta, while the expression of SNX16 is increased (Zhang L. et al., 2013). Up-regulation of miR-196a-5p can inhibit SNX16 expression. Luciferase reporter gene analysis has shown that miR-196a-5p binds to the 3′ UTR of SNX16 mRNA. An increase of miR-196a-5p or knockdown of SNX16 can restore cell viability, migration, invasion, and expression of matrix metalloprotein 2 (MMP-2) and MMP-9 under hypoxic conditions. Furthermore, BHLHE40 binds to the promoter of the miR-196a-5p gene. BHLHE40 knockdown alleviated PE symptoms in pregnant C57/BL6N mice, suggesting that the BHLHE40/miR-196a-5p/SNX16 axis is involved in the pathogenesis of PE (Zhang L. et al., 2013).

Among hypoxia-related miRNAs, miR-141 (Wu D. et al., 2019), miR-517 (Anton et al., 2015), miR-218, and their host genes slit guidance ligand 2 (SLIT2) and SLIT3 have elevated expression levels in placental tissues of patients with PE (Fang et al., 2017). miR-141 down-regulates the expression of MMP-2, p62, and microtubule-associated protein 1A/1B-light chain 3 (LC3B) in HTR-8/SVneo cells by targeting the C-X-C motif chemokine ligand 12β (CXCL12β) gene, while also up-regulating Rho-associated coiled-coil containing protein kinase 1 (ROCK1) and Ras homolog family member A (RhoA) expression, promoting the apoptosis of HTR-8/SVneo cells and inhibiting their invasion and vascularization abilities (Wu D. et al., 2019). In vitro cell experiments indicate that hypoxia can induce high expression of miR-218, which is down-regulated after HIF-1α gene knockout. Chromatin immunoprecipitation results suggest that HIF-1α can directly bind to the promoters of SLIT2 and SLIT3. Further studies have shown that miR-218 targets LIM and SH3 protein 1 (LASP1), thereby inhibiting the infiltration of trophoblast cells, which may eventually induce the onset of PE (Fang et al., 2017). Hypoxia affects the expression of miR-145-5p, which is down-regulated in the PE placenta. Low expression of miR-145-5p inhibits the activity and invasiveness of HTR-8/SVneo cells, whereas overexpression of miR-145-5p promotes these effects. Furthermore, research suggests that FLT1 is the target gene of miR-145-5p (Lv et al., 2019).

#### Regulation of Angiogenesis Factors and Promotion of Vascular Endothelial Damage

Angiogenesis plays a key role in tissue repair and organ growth, and an imbalance of angiogenesis can easily lead to the occurrence and development of PE (Zhang M. et al., 2016). The dynamic balance between vasodilator and contractile factors, such as VEGF and its receptor sFlt-1 (Zhu et al., 2009), PLGF (secreted by placental trophoblasts), and endothelin, can promote the growth, differentiation, migration, and infiltration of blood vessels in the placenta and decidua, and maintain vascular intima integrity and placental vascular permeability.

miR-126 is a powerful miRNA related to angiogenesis. miR-126 expression decreases in pregnant women with PE, which is positively correlated with the degree of placental ischemia in PE. Blood flow can activate zinc finger transcription factors to induce miR-126 expression (Yan et al., 2013). An imbalance of angiogenic factors in PE, including a decrease of pro-angiogenic factor VEGF-A and increase in hypoxia, lead to reductions of miR-126 expression and endothelial cell numbers (Yan et al., 2013). Hong et al. (2014) showed that miR-126 was under-expressed in the placenta tissues of patients with PE based on quantitative reverse transcription polymerase chain reaction (qRT-PCR), and was positively correlated with VEGF mRNA expression. In addition, miR-126 and miR-378 target tumor suppressor candidate 2 (TUSC2) to up-regulate pro-angiogenic factors; therefore, low expression levels of these two miRNAs in PE may lead to spiral artery remodeling disorder, placental ischemia, hypoxia, and hypoperfusion (Weber et al., 2016). Studies on the pathway of miR-126/PIK3R2/PI3K/AKT cells indicate that the 3′ UTR of miR-126 targets and regulates phosphoinositide-3-kinase regulatory subunit 2 (PIK3R2), which is an important component of the VEGF pathway. After miR-126 activation, PIK3R2 expression decreases, while the mRNA and protein levels of phosphoinositide 3-kinase (PI3K) and protein kinase B (AKT) increase (Zhu et al., 2009), which further illustrates the important role of miR-126 that promotes angiogenesis and anti-apoptotic mechanisms, suggesting its potential function in the pathogenesis of PE.

The survival of mesenchymal stem cells (MSCs) and angiogenesis at the maternal-fetal interface are essential factors for a successful pregnancy. miR-136 is highly expressed in MSCs derived from PE decidua. High levels of miR-136 can inhibit the proliferation of MSCs and promote the apoptosis of MSCs by targeting BCL2, and can impair human umbilical vein endothelial cell (HUVEC) capillary formation and trophoblast cell invasion by inhibiting VEGF (Ji et al., 2017). In addition, the expression level of miR-29b increases in decidua-derived mesenchymal stem cells (dMSCs) of severe PE patients, and overexpression of miR-29b targets histone deacetylase 4 (HDAC4) to reduce the proliferation ability of dMSCs, as well as the migration and tubule-formation abilities of HUVECs (Xin et al., 2017). Expression of miR-495 increased in the umbilical cord tissue and MSCs of patients with pregnant women with severe PE (Li et al., 2017b). Further studies confirmed that miR-495 targets BMI-1 to promote cell apoptosis, inhibit the migration and invasion of trophoblasts, and hinder tubule formation by HUVECs.

#### Roles in Trophoblast Cell Invasion

Trophoblasts are the main cell type of the placenta and are involved in the formation of the placenta. miR-210 not only influences trophoblast invasion by adapting to oxidative stress but also directly affects cell invasion by targeting specific cell invasion markers. In PE placentas, the thrombospondin type 1 domain containing 7A (THSD7A) level is significantly down-regulated and inversely proportional to the level of miR-210. Hypoxia significantly increases miR-210 expression and inhibits THSD7A expression in a time-dependent manner, confirming that THSD7A inhibits the invasion of miR-210 into HTR8/SVneo cells (Luo et al., 2016). In another study, up-regulation of miR-210 reduced Notch1 expression; impaired HTR8/SVneo proliferation, migration, invasion, and tubule formation; and promoted apoptosis; down-regulation of miR-210 had the opposite effects. These findings suggest that miR-210 plays a role in trophoblast dysfunction by reducing Notch1 expression (Wang R. et al., 2019).

As shown by microarray analysis, miR-49 is highly expressed in dMSCs in PE. miR-494 prevents the G1/S transition of dMSCs by targeting cyclin dependent kinase 6 (CDK6) and cyclin D1 (CCND1). The supernatant of miR-494-overexpressing dMSCs reduces the migration of HTR-8/SVneo cells and impedes the formation of HUVEC capillaries by inhibiting VEGF (Chen S. et al., 2015). In situ hybridization demonstrated that the level of miR-20a was higher in PE than in normal placental tissue, and showed that the up-regulation of miR-20a can inhibit the proliferation, migration, and invasion of trophoblast cells by inhibiting the expression of forkhead box A1 (FOXA1) (Wang Y. et al., 2014). In addition, miR-646 expression is elevated in endothelial progenitor cells isolated from the peripheral blood of pregnant women with PE (Dong D. et al., 2020). miR-646 reduces angiogenesis and inhibits the proliferation, differentiation, and migration of endothelial progenitor cells by down-regulating VEGF-A and HIF-1α expression.

#### Roles in Immune Regulation and Inflammation

As a powerful tool for post-transcriptional regulation, microRNAs not only participate in the pathological process of PE through oxidative stress, hypoxia damage, vascular endothelial damage, and regulating the biological functions of trophoblast cells, but also regulate the immune response. In a healthy pregnancy, proper immune regulation prevents excessive systemic inflammation. In PE, abnormal activation of decidual T cells, uterine natural killer cells, macrophages (Faas and De Vos, 2018), and the imbalanced differentiation of helper T cell subsets destroy normal maternal-fetal immune tolerance. The activation of immune cells and subsequent secretion of cytokines affect angiogenesis, trophoblast invasion, and spiral artery remodeling, which is considered to be an indirect cause of insufficient reconstruction of the uterine spiral artery (Geldenhuys et al., 2018). MiR-223 was down-regulated in the placenta and circulating samples of pregnant women with PE (Sheikh et al., 2016). Shi et al. (2013) showed that after miR-223 knockout, the expression of immune and inflammation-related genes in mice changes significantly, with the number of immune T cells decreasing and the fetus-to-placenta mass ratio gradually decreasing as pregnancy progresses. After the female mouse reproductive tract comes into contact with semen, the expression of miR-223 is up-regulated. In contrast, down-regulation of miR-223 expression has an adverse effect on immune tolerance in female mice. Therefore, after maternal exposure to semen containing foreign antigens, miR-223 can increase maternal immune tolerance and foster pregnancy. In addition, studies of the IL-6/miR-223/STAT3 cell pathway suggest that down-regulation of miR-223 expression can lead to activation of signal transducer and activator of transcription 3 (STAT3), which is accompanied by pro-inflammatory production of the cytokine interleukin 6 (IL-6). Enhanced IL-6 expression can reduce miR-223 levels, leading to vascular dysfunction and promoting macrophage infiltration, with adverse effects on the invasion of cytotrophoblast cells into the decidua and myometrium (Lockwood et al., 2008). Immunohistochemistry has further demonstrated that the decidua and trophoblast cells of patients with PE exhibit high expression of IL-6 (Lockwood et al., 2008), which is the main factor inducing decreased miR-223 expression Dong D. et al. (2020). Low expression of miR-223 in PE may be a compensatory mechanism that activates endothelial function and increases angiogenesis. Down-regulation of miR-223 increases the expression of β1 integrin and promotes angiogenesis (Shi et al., 2013). Ischemia and hypoxic stress can target the upstream promoter of miR-223 CCAAT enhancer binding protein alpha (C/EBP-α) through HIF protein, thereby reducing the miR-223 level (Seifeddine et al., 2008).

Inflammation-related miR-125b is up-regulated in placental tissues and serum of PE patients. Sphingosine-1-phosphate lyase-1 (SGPL1) is a target gene of miR-125b. miR-125b down-regulates SGPL1 expression and enhances IL-8 production, which can be reversed by abolishing the overexpression of SGPL1. Therefore, miR-125b can increase IL-8 expression in PE, which in turn induces maternal inflammation (Yang et al., 2016). Kopriva et al. (2013) suggested that high expression of miR-210 in the placenta of patients with PE is related to inflammatory factors, and that the appearance of inflammation is related to overactivation of toll-like receptor 3 (TLR3) (Bugge et al., 2017). TLR3 is activated in patients with PE (Chatterjee et al., 2012); by activating HIF-1α and NF-κBp50, TLR3 induces the up-regulation of miR-210 and further down-regulates the target gene signal transducer and STAT6. miR-155 can enhance the AP-A/NK-kB inflammatory response pathway by regulating cyclin D1, which targets interleukin 1 receptor associated kinase 3 (IRAKM), NFKB inhibitor interacting Ras like 1 (NKIRAS1), and phosphatase and tensin homolog (PTEN), suggesting that miR-155 is related to superficial implantation in spiral arteries, a characteristic of PE (Xue et al., 2013).

In general, Each miRNA has multiple target genes, such as miR-210, which not only targets HSD17B1s (Song et al., 2015), but also targets PTPN2 (Li J. et al., 2019), KCMF1 (Luo et al., 2014), THSD7A (Tian et al., 2020), and Notch1 (Wang R. et al., 2019) to regulate the biological function of trophoblast and participate in vascular remodeling, growth and tube formation. Besides, several miRNAs also target the same gene. MMPs are important regulators of vascular and uterine remodeling. Decreased vascular MMP-2 may lead to the decrease of vasodilation, the enhancement of vasoconstriction, hypertensive pregnancy, and PE (Chen and Khalil, 2017). miR-141 (Wu D. et al., 2019) and miR-520g (Jiang et al., 2017) down-regulate the expression of MMP-2 and participate in the pathogenesis of PE by inhibiting angiogenesis and the migration, invasion, proliferation of trophoblast. This complex regulatory network not only regulates the expression of multiple genes through one miRNA, but also fine-tunes the expression of specific genes through cooperation of several miRNAs.

## LncRNA in PE

### LncRNA

Long non-coding RNA is commonly present in eukaryotic cells. Defined by a length of more than 200 nucleotides and lack of open reading frames, lncRNA can regulate gene expression at the epigenetic, transcriptional, and post-transcriptional levels (Sun and Kraus, 2015), and its proportion exceeds 80% of total RNA (Guttman and Rinn, 2012). Most lncRNAs are produced through transcription by RNA polymerase II, and their sources are diverse. They can be produced due to the destruction of gene structures encoding proteins, or through rearrangement of two separate untranslated chromosomes to produce a lncRNA containing multiple exons. According to the genomic location of lncRNAs, they can be roughly divided into five categories: sense lncRNA, antisense lncRNA, intragenic lncRNA, intergenic lncRNA, and bidirectional lncRNA. lncRNAs are important cis- and trans-regulators of gene activity, but their expression is primarily cis-acting, often reflecting the expression of neighboring genes. The expression and function of lncRNAs have tissue and spatiotemporal specificities, and play important roles in many life processes, such as chromatin modification, dose compensation effects, epigenetics, cell cycle, cell differentiation, transcription, and post-transcriptional regulation (Delas and Hannon, 2017). Their main regulation methods include acting as an activator or inhibitor of the transcript via coordinated interactions with the RNA-binding protein or regulation of the promoter of the targeted protein; forming an RNA-DNA complex to prevent the binding of transcripts; regulating DNA methylation or modifying histones; and forming complexes with proteins that affect the positioning of those proteins. lncRNA can also act as a miRNA sponge through base pairing and formation of the RISC, which reduces the activities of these small RNA molecules and inhibits the expression of miRNA-targeted genes (Guo et al., 2014).

### LncRNAs in the Pathogenesis of PE

Long non-coding RNA have emerged as critical molecular regulators in biological processes and diseases, such as cancer, Alzheimer’s disease, and cardiovascular disease. Recently, many groups have indicated that abnormally expressed lncRNAs in the placenta and circulating blood of pregnant women with PE participate in the pathophysiological process of PE by regulating the biological functions of trophoblasts, angiogenesis, decidualization, and inflammation. Table 2 shows the differential expression of lncRNAs involved in PE pathophysiology.

**TABLE 2:** LncRNAs in the pathogenesis of Preeclampsia.

lncRNAs	Source	Expression	Target	Function	References
AGAP2-AS1	Placental tissues	downregulated	miR-574-5p/JDP2	proliferation, invasion and apoptosis	Xu et al., 2020
ATB	Placental tissues	downregulated	-	migration, proliferation, and tube formation	Liu X. et al., 2017
CCAT1	Placental tissues	upregulated	D1-P16-CDK4	cell proliferation and cell cycle	Deng et al., 2015
DC	Dendritic cells	upregulated	p-STAT3	invasion	Zhang W. et al., 2017, 2020
EGFR-AS1	Placental tissues	downregulated	EGFR-JAK/STAT	proliferation	Zhao and Jiang, 2018
H19	Placental tissues	upregulated	PI3K/AKT/mTOR	cell viability, invasion, autophagy	Xu J. et al., 2018
	Exosomes secreted by MSCs	upregulated	microRNA let-7b/FOXO1	invasion, migration and apoptosis	Chen Y. et al., 2020
HOTAIR	Placental tissues	upregulated	miR-106a/EZH2	proliferation, migration and invasion	Zhao et al., 2020
HOTTIP	Placental tissues	downregulated	RND3	cell proliferation and cell cycle	Li et al., 2018
KCNQ1OT1	Placental tissues	downregulated	miR-146a-3p/CXCL12/CXCR4	proliferation, invasion and migration	Chen Y. et al., 2020
Linc00261	Placental tissues	upregulated	miR-558/TIMP4	invasion, migration and apoptosis	Cheng et al., 2019b
LINC00511	Placental tissues	downregulated	miR-31-5p/HOXA7	proliferation, invasion and autophagy	Dong X. et al., 2020
lncRNA uc.187	Placental tissues	upregulated	-	proliferation, invasion and apoptosis	Cao et al., 2017
lncZBTB39	Placental tissues	upregulated	miR-210/THSD7A	invasion and migration	Tian et al., 2020
MALAT1	Placental tissues	downregulated	-	proliferation, migration invasion and apoptosis	Chen H. et al., 2015
	Placental tissues	downregulated	FOS	EMT, migration and invasion	Li et al., 2020
	Placental tissues	downregulated	miR-206/IGF-1	migration and invasion	Wu et al., 2020
	Umbilical cord tissues and MSCs	downregulated	VEGF/IDO	proliferation, angiogenesis, and Immunosuppressive	Li et al., 2017a
MEG3	Placental tissues	downregulated	NF-kB, Caspase-3, and Bax	apoptosis and migration	Zhang et al., 2015; Liu W. et al., 2017
MIR503HG	Placental tissues	upregulated	NF-kB, MMP-2/-9, the snail protein, and E-cadherin	proliferation, invasion, migration, apoptosis and cell cycle	Cheng et al., 2019a
NR_002794	Placental tissues	upregulated	-	proliferation, invasion and apoptosis	Ma et al., 2019
PGK1P2	Placental tissues	downregulated	miR-330-5p	angiogenesis, glycolysis metabolism and decidualization	Tong et al., 2018
PVT1	Placental tissues	downregulated	PI3K/AKT pathway	proliferation, migration and invasion	Xu Y. et al., 2018; Wang Q. et al., 2019
RP11-465L10.10	Placental tissues	downregulated	MMP-9	invasion	Fan et al., 2016; Long et al., 2016
RPAIN	Placental tissues	upregulated	C1q	proliferation, invasion and apoptosis	Song et al., 2017
SNHG5	Placental tissues	downregulated	miR-26a-5p/N-cadherin	proliferation, migration and invasion	Yang et al., 2019
SPRY4-IT1	Placental tissues	upregulated	Wnt/β-catenin pathway	proliferation, migration and apoptosis	Zou et al., 2013, Zuo et al., 2016
Storkhead box 2	Placental tissues	upregulated	STOX2	differentiation and invasion	Oudejans et al., 2016
TCL6	Placental tissues	upregulated	PTEN	cell proliferation and cell cycle	Wu J.L. et al., 2019
TDRG1	Placental tissues	downregulated	miR-214-5p, Notch	proliferation, migration, and invasion	Gong et al., 2020b
TUG1	Placental tissues	downregulated	miR-204-5p, miR-29b/MCL1/VEGFA/MMP-2	migration, invasion, proliferation, apoptosis and angiogenesis	Yu et al., 2018; Li Q. et al., 2019
ZEB2-AS1	Placental tissues	downregulated	miR-149/PGF	proliferation, migration and invasion	Gao et al., 2019

#### Roles of lncRNAs in Regulating the Invasion and Migration of Trophoblast Cells

Trophoblast cells have invasion and migration characteristics like tumor cells. Insufficient infiltration of placental trophoblast cells and poor vascular remodeling are associated with pregnancy-related complications, including recurrent miscarriage, PE, eclampsia, and intrauterine growth retardation. lncRNA MALAT-1 was firstly identified in lung cancer (Ji et al., 2003). The expression level of MALAT1 lncRNA was reduced in the placentas of PE patients. After silencing of MALAT1 in the trophoblast cell line JEG-3, the cell cycle was arrested in the G0/G1 phase; caspase 3, caspase 9 and poly(ADP-ribose) polymerase 1 (PARP-1) levels increased significantly; and cell invasion and migration were inhibited (Chen S. et al., 2015). Similarly, Li et al. (2020) observed low MALAT1 expression in the placenta of early-onset eclampsia patients. Based on the RNA sequence, Fos was identified as a downstream functional gene of MALAT1. MALAT1 promotes the migration and invasion of trophoblast cells via Fos-induced EMT. Silencing the expression of MALAT1 (-/-) inhibits the invasion and migration of trophoblast cells. MALAT1-/- also leads to reduced expression of N-cadherin and vimentin, while the expression of E-cadherin is elevated, highlighting the role of MALAT1 in spiral artery remodeling damage during the placental pathogenesis of early eclampsia. Another study demonstrated that MALAT1 expression decreased in the placenta of pregnant women with PE, whereas miR-206 expression increased. A dual-luciferase reporter experiment verified the interactions of MALAT1 and miR-206, as well as miR-206 and insulin-like growth factor-1 (IGF-1). MALAT1 inhibits trophoblast cell migration and invasion by regulating the IGF-1/PI3K/Akt signaling pathway via miR-206 (Wu et al., 2020). MALAT-1 plays a key role in regulating the angiogenesis, vascularization of the maternal decidua and fetal umbilical vasculature. Expression of MALAT1 in umbilical cord tissues and MSCs of patients with severe PE are also reduced (Li et al., 2017a). Transfection of a MALAT1 plasmid into MSCs causes the cell cycle to enter the G2/M phase and inhibits apoptosis. The supernatant of MALAT1-overexpressing MSCs promotes MSC migration, HTR-8/SVneo cell invasion, and HUVEC tube formation, whereas silencing of MALAT1 has the opposite effects. Hydrogen peroxide reduces MALAT1 expression in MSCs in a dose-dependent manner, indicating that peroxide may cause down-regulation of MALAT1 expression (Li et al., 2017a). In general, low expression of MALAT1 in the placenta of PE affects the cell cycle, apoptosis, invasion, migration, spiral artery remodeling, and tube formation through different target molecules.

Long non-coding RNA KCNQ1 is an imprinted gene that expresses only paternal alleles. KCNQ1OT1 mRNA expression was reduced in PE placental tissues, while miR-146a-3p expression was increased. A dual-luciferase reporter gene experiment confirmed the targeting relationship between KCNQ1OT1 and miR-146a-3p. Silencing KCNQ1OT1 expression up-regulates miR-146a-3p expression; inhibits HTR8/SVneo cell proliferation, invasion, and migration; and prevents CXCL12/CXCR4 signaling pathway activation. Thus, inhibiting miR-146a-3p expression can attenuate KCNQ1OT1, regulating cellular proliferation, invasion, and migration abilities and inhibiting the CXCL12/CXCR4 signaling pathway. The KCNQ1OT1/miR-146a-3p/CXCL12/CXCR4 molecular system may act as a regulatory network in the pathogenesis of PE by affecting the function of trophoblast cells (Chen F.R. et al., 2020). Yu et al. (2018) found that lncRNA TUG1 was significantly down-regulated in PE placental tissue, which inhibited the migration and invasion of trophoblast-like cells. Bioinformatics and functional analysis show that TUG1 interacts with miR-204-5p and negatively regulates the expression and function of miR-204-5p in trophoblast cells. TUG1 regulates the migration and invasion of trophoblasts through sponging of miR-204-5p; therefore, the TUG1/miR-204-5p axis may be a potential target for the intervention of PE. In another study, TUG1 was significantly reduced in the placentas of PE patients. Ectopic expression of TUG1 promotes cell proliferation, invasion, and angiogenesis, but has a negative regulatory effect on cell apoptosis. TUG1 can act as a molecular sponge of miR-29b, thereby regulating MCL1, VEGF-A, and MMP-2 during the development of PE (Li Q. et al., 2019). In addition, lncZBTB39 is up-regulated in the PE placenta, and its overexpression (downregulation) inhibits (enhances) invasion and migration of HTR8/SVneo cells and MMP-2 activity. lncZBTB39 negatively regulates the invasion and migration of trophoblast cells, and likely maintains THSD7A mRNA expression through sponging of miR-210 (Tian et al., 2020).

The expression of lncRNA SPRY4-IT1 is elevated in PE placenta, which affects the EMT of vegetative cells via the Wnt/β-catenin signaling pathway, thereby inhibiting trophoblast invasion and migration (Zuo et al., 2016). Similarly, lncRNA STOX2-IT3 regulates STOX2 gene expression to attenuate the differentiation and invasion of trophoblasts (Oudejans et al., 2016). Song et al. (2017) reported that lncRNA RPAIN is highly expressed in the placentas of patients with PE. When RPAIN is overexpressed, trophoblast proliferation and invasion is significantly inhibited, and trophoblast apoptosis increases significantly. Overexpression of RPAIN inhibits the expression of the complement protein C1q. C1q overexpression rescues the reduced cell invasion and enhanced apoptosis of trophoblast cells overexpressing RPAIN. Elevated RPAIN levels may play an important role in trophoblast invasion and spiral artery recasting via C1q regulation (Song et al., 2017).

#### LncRNAs Inhibit Trophoblast Proliferation and Cell Cycle

The colon cancer-related transcription factor 1 (CCAT1) gene is a lncRNA that is abnormally expressed in colon cancer tissues and is associated with proliferation activity and metastasis of colon cancer cells (Zhu et al., 2015). Higher expression of lncRNA CCAT1 was found in patients with PE. PE patients were divided into high-expression and low-expression groups according to CCAT1 expression. Compared with the low-expression group, the systolic blood pressure, diastolic blood pressure, and urine protein levels of CCAT1 high-expression group were elevated, while birth weight was significantly higher in the low-expression group than the high-expression group. In addition, overexpression of CCAT1 to observe the function of CCAT1 in the proliferation activity and cell cycle of Bew and JEG-3 cells showed that interference with CCAT1 led to significant trophoblast proliferation and cell cycle acceleration, whereas overexpression of CCAT1 had the opposite results. After overexpression of CCAT1, the expression levels of E2F1, cyclin D, CDK2, and CDK4 in JEG3 cells were significantly reduced, while inhibition of CCAT1 led to significantly increased expression of E2F1, cyclin D, CDK2, and CDK4 in BeWo cells, suggesting that CCAT1 may be involved in the regulation of placental trophoblasts in PE, and thus play a role in the pathogenesis of PE (Deng et al., 2015).

#### LncRNAs Regulate Angiogenesis and Decidualization

Impaired decidual function can lead to failure of trophoblast infiltration into blood vessels and insufficient placental function, thereby increasing the risk of PE. The expression level of lncRNA SPRY4-IT1 is 2.8 times higher in the placentas of PE patients than in normal pregnancy. Silencing of SPRY4-IT1 with small interfering RNA (siRNA) leads to an increase in its migration phenotype and a decrease in cell apoptosis. At the same time, siRNA silencing of SPRY4-IT1 results in decreased tube formation ability, while overexpression of SPRY4-IT1 enhances tube formation (Zou et al., 2013). Knockout of SPRY14-IT1 leads to decreased gene and protein expression levels of E-cadherin and β-catenin, as well as increased vimentin expression. The mechanism of those effects may be the binding of SPRY14-IT1 to the cytoplasmic RNA-binding protein HuR, and the resulting complex directly binding to β-catenin mRNA (Zuo et al., 2016). The interaction between HuR and β-catenin proteins leads to an imbalance in β-catenin mRNA expression, which has been verified in other cell types (Chou et al., 2015). Knockdown of SPRY14-IT1 can lead to down-regulation of Wnt3 and Wnt5B, the downstream targets of β-catenin. Strict regulation of Wnt/β-catenin signaling plays important roles in maintaining the phenotype of epithelial cells and the development of cell-cell connections. The loss of this pathway leads to type 1 EMT. The EMT of extravillous trophoblasts is a key step in the invasion of maternal decidua tissue, and is essential to uterine spiral artery recasting and successful pregnancy (Gude et al., 2004). Expression of SPRY4-IT1 is associated with the formation of an endothelial cell-like tubular network, which is an important process in angiogenesis; in particular, high expression of SPRY4-IT1 in the placenta of patients with PE may be related to the process of uterine spiral artery recasting.

Phosphoglycerate kinase 1 (PGK1) is an enzyme involved in the glycolytic pathway, which is the main metabolic process undertaken by DCs. The sequences of lncRNA PGK1P2 and PGK1 are similar. Normal decidualization is essential for normal pregnancy. In one study, PGK1 and PGK1P2 mRNA expression and PGK1 protein level were lower in decidua of severe PE than in the normal pregnancy control group. Further research showed that PGK1 and PGK1P2 are a pair of ceRNAs targeting miR-330-5p. They play a crucial role in human decidualization by regulating angiogenesis and glycolytic metabolism. Lack of PGK1 and PGK1P2 in the decidua inhibits the decidualization process and may subsequently lead to PE (Tong et al., 2018).

#### LncRNAs Participate in Inflammation and Immune Regulation

LncRNA MALAT1 is also involved in immune regulation and inflammation. MALAT1 reduces the secretion of inflammatory factors TNF-α and IL-6, and inhibits the expression of TGF-β and NF-κB in trophoblastic cells (Zhang Y. et al., 2020). lncRNA H19 plays role in inflammatory diseases (Wang W.T. et al., 2016). Excessive inflammation of maternal endothelial cells is a characteristic of PE (Chen et al., 2016). The placental tissues of PE patients have higher levels of lncRNA H19 than healthy controls. Overexpression of lncRNA H19 activates PI3K. The PI3K/AKT/mTOR pathway reduces cell viability and promotes autophagy in trophoblasts, but increases cell invasion (Xu J. et al., 2018). lncRNA DC is expressed exclusively in human DCs and induces DC differentiation and maturation by promoting the phosphorylation of STAT3 (Wang P. et al., 2014). lnc-DC and p-TAT3 increase in PE, and overexpression of lnc-DC inhibits trophoblast invasion, increases p-STAT3 levels, and increases the ratios of tissue inhibitor of metalloproteinase (TIMP)-1 to MMP-9 and TIMP-2 to MMP-2 (Zhang W. et al., 2020). In addition, overexpression of lnc-DC can cause excessive maturation of DCs in patients with PE and increase the abundance of Th1 cells, indicating that lncRNA DC has immunomodulatory activity (Zhang W. et al., 2017).

## CircRNAs in PE

### CircRNA

Circular RNA is a closed circRNA with no 5′ cap and with a 3′ poly-A tail (Memczak et al., 2013). The sequence of circRNA is highly conserved, and its circular structure resists degradation by exonucleases. Its structure is relatively stable and highly expressed, and it plays roles in gene transcription and post-transcriptional regulation (Memczak et al., 2013). The length of circRNAs is in the range of 200–2,000 bp, and most are about 500 bp. circRNA is a type of endogenous RNA that is transcribed by RNA polymerase II (Zhang et al., 2018), and can be divided into non-coding and coding circRNAs. Coding circRNA has an open reading frame that generally contains at least one internal ribosome entry site (Li et al., 2019c). The closed-loop structure can be formed through the reverse splicing mechanism, wherein the upstream 5′ splice site and downstream 3′ splice site are connected by linear mRNA; therefore, canonical spliceosome signaling may be involved in the regulation of circRNA biogenesis (Chen, 2016). circRNA expression is tissue-specific, and diverse types of circRNA are expressed (Xia et al., 2017). According to the source of splicing, circRNA can be divided into exon source, intron source, or exon and intron source types (Han et al., 2018). Most circRNAs are derived from exons, and a small number are formed through direct circularization of introns. The main biological functions of circRNA are as follows. As a miRNA sponge, circRNA is rich in miRNA-binding sites and can function as ceRNA. circRNA can absorb miRNA through sponging or base complementation and inhibit its binding to mRNA, increasing mRNA expression (Han et al., 2018). By participating in the regulation of linear splicing (Hansen et al., 2013), it functions alongside RNA polymerase II as a cis-acting element and regulatory gene in transcription (Zhang Y. et al., 2013). Moreover, circRNA can interact with RNA-binding proteins as a scaffold to promote, align, and regulate RNA functions (Memczak et al., 2013) play roles in transcription and translation (Pamudurti et al., 2017), and act as an m6A recognition protein, known as YTH N6-methyladenosine RNA binding protein 3 (YTHDF3). It can bind to the modified start site of circRNA and recruit translation initiation factors such as eIF4G2 for translation (Yang et al., 2017).

### CircRNAs in the Pathogenesis of PE

As a novel type of ncRNA, circRNA has notable advantages for development and application as a clinical biomarker. Although few studies on circRNA in the pathogenesis of PE have been conducted, it has been revealed that circRNA in the plasma and placenta tissue of patients with PE can block the inhibitory effect of miRNA on its target mRNA by binding to miRNA, thereby regulating mRNA expression. This mechanism may allow circRNA to participate in the pathogenesis of PE.

Deng et al. (2019) integrated RNA sequencing data from human placentas with severe PE and normal pregnancies, and found that 180 circRNAs were differentially expressed in placentas with severe PE, including 94 up-regulated and 86 down-regulated circRNAs. Most of the detected circRNAs have not been annotated. Bioinformatics analysis of differentially expressed circRNAs shows that most are involved in vasodilation, blood vessel size regulation, protein transport and localization, and cancer pathways. circRNA mainly acts in a regulatory role as a sponge of miRNA. ceRNA inhibits its binding to mRNA and increases mRNA expression. The circ_0085296 level was elevated in PE placental tissue, while miR-144 was down-regulated (Zhu et al., 2020). Up-regulation of circ_0085296 inhibits trophoblast proliferation, invasion, and migration. miR-144 directly binds to circ_0085296 and E-cadherin, and circ_0085296 acts as a sponge of miR-144 to regulate the expression of E-cadherin. Moreover, miR-144 inhibition and E-cadherin overexpression weaken the effect of circ_0085296 on cellular processes in trophoblast cells. hsa_circ_011277 (Ou et al., 2020), hsa_circ_0011460 (Deng et al., 2019), and hsa_circ_0006772 (Shen et al., 2019) are significantly elevated in the placenta of PE patients. circ_011277 reduces the inhibitory effect of miR-494-3p by regulating the expression of HtrA serine peptidase 1 (HTRA1) and Notch1 (Ou et al., 2020), while hsa_circ_0011460 directly targets the solute carrier organic anion transporter family member (PGT) interaction (Deng et al., 2019). hsa_circ_0006772 has a negative regulatory effect on trophoblast migration and EMT. Meanwhile, the hsa_circ_0006772 sponge miR-762 inhibits the activity of miR-762 and increases the protein level of the EMT-related transcription factor grainyhead like transcription factor 2 (Grhl2) (Shen et al., 2019). hsa_circ_0000284 (Zhang Y. et al., 2019) and hsa_circ_0003286 (Zhou et al., 2018) were reduced in PE placental tissue. After siRNA transfection to silence hsa_circ_0000284, the migration, invasion, proliferation, and tube formation activities of HTR8/SVneo cells were inhibited, while siRNA transfection silencing hsa_circ_0003286 significantly reduced cell invasion.

Expression of hsa_circ_0036877 in the placenta was lower in pregnant women with PE than in normal pregnant women, but that in peripheral blood was higher in pregnant women with PE than in normal pregnant women (Hu et al., 2019). hsa_circ_0036877 combines miR-519d-3p and miR-15b-5p, and may act as a miRNA sponge in the pathogenesis of PE (Hu et al., 2018). Syncytial trophoblast cell necrosis is a direct result of unbalanced regulation of apoptosis, and leads to increased release of trophoblast granules into the circulating blood of patients with PE (Huppertz et al., 2006). hsa_circ_0036877 may be an extracellular nucleic acid derived from the placenta. The marked increase in apoptosis of syncytial trophoblast cells may explain the low expression of hsa_circ_0036877 in the PE placenta but high expression in circulating blood.

Liu et al., 2019 analyzed differentially expressed circRNA, lncRNA, miRNA, and mRNA based on RNA sequencing data from placenta tissues and data in the Gene Expression Omnibus (GEO) database using gene set enrichment analysis and other bio-informatics methods. The results showed that the JAK/STAT signaling pathway was activated and miR-100-5p was down-regulated in both PE sequencing data and GEO data, and the expression levels of lncRNA-1492, hsa_circ_0088196, and leukemia inhibitory factor (LIF), which have targeted regulatory relationships with miR-100-5p, were up-regulated. A luciferase reporter experiment demonstrated the presence of a ceRNA regulatory network in PE, wherein lncRNA-1492 and hsa_circ_0088196 target the inhibitory effect of miR-100-5p on LIF, and up-regulated LIF activates the JAK/STAT pathway, promoting the development of PE.

During early pregnancy before the onset of PE, prediction, effective intervention, prevention, and control of the disease are of great significance (Lavallee, 2015). circRNA has characteristics of stability, universality, specificity, and conservation, making it a more suitable biomarker than other RNA types (Lasda and Parker, 2014). A prospective study of circRNA expression in the blood cells of PE patients before 20 weeks of pregnancy and healthy pregnant women showed that PE patients had significantly higher hsa_circ_101222 (Zhang Y.G. et al., 2016). Analysis using bioinformatics prediction (Liu et al., 2019) indicated that hsa_circ_101222 may interact with miR-181, which is closely related to the pathogenesis of PE (Liu et al., 2012), while the miRNA response elements of hsa_circ_0001855 and hsa_circ_0004904 were identified as miR-138-5p, miR-30c-1-3p, miR-623, miR-30c-2-3p, miR-134-3p, miR-29a-5p, and miR-765, all of which target pregnancy-associated plasma protein A (PAPP-A), and miR-29a-5p, and are closely related to the pathogenesis of PE (Stubert et al., 2014). The area under the receiver operating characteristic curve for the two circRNAs combined with PAPP-A can reach 0.940 (95% confidence interval: 0.869–1.000), suggesting that hsa_circ_0004904 and hsa_circ_0001855 in combination with PAPP-A are promising biomarkers for the prediction of PE.

## Conclusion

Preeclampsia is asymptomatic in the early stage, so early diagnosis is essential for PE. To identify potential biomarkers of PE, many groups focused on non-coding RNAs in maternal plasma and serum. As regards miRNAs and lncRNAs in maternal blood, there are still no sufficient data to understand if they can be used as promising markers for PE monitoring and screening, since almost all of the case-control designs only considered the affected cases and the unaffected cases. Besides, the population recruited by PE is heterogeneous, including variation in the definition of PE and the timing of screening during pregnancy, so the possible independent positive predictive value of miRNAs and lncRNAs is unclear. Additionally, abnormal expression of miRNAs and lncRNAs is only found in symptomatic PE cases. Because the true incidence is not provided, miRNAs and lncRNAs are currently useless for the possible implementation of screening purposes. Considering the molecular advantages of circRNAs, studies on circRNA are more focused on possible use for screening purposes, and are generally better designed, as they consider a wider number of cases. The first study to report circRNA analysis in pregnant women with PE before the onset of symptoms found that hsa_circ_0004904 and hsa_circ_0001855 were significantly elevated in pregnant women with PE compared with healthy pregnant women. The area under the curve of the combined model was 0.94 in the predicted PE subject (Jiang et al., 2018). Unfortunately, even though some papers reported the possible screening performance of circRNAs at the first or second trimester of pregnancy, circRNAs have not been used successfully for any screening program for PE considering the small cohort of patients studied. Further prospective studies with larger samples from different gestational stages are needed to understand the possible role of circRNAs in predicting and monitoring PE. The abnormal expression of PLGF, PAPP-A, PP13, and sFlt-1 in the pathological process of PE has potential value in the prediction of PE. In addition to the above factors, there are more biological indicators in the clinical application value of PE that have been found. Considering the economy and efficiency of clinical application, multi-index combined prediction, including PAPP-A, PP13, sFlt-1, circRNAs, and other biological indicators, combined with maternal history, ultrasound, and so on, may have a greater application prospect.

The pathogenesis of early- and late-onset PE is different, and the role of non-coding RNAs in them may also be different. Deep sequencing compared the expression profiles of tissue and circulating miRNAs in pregnant women with early- and late-onset PE, and found differential expression of miRNA in the two groups of tissues (Timofeeva et al., 2018). In early-onset PE, miR-431, miR-518a-5p, and miR-124 were up-regulated in the placenta tissues (Lykoudi et al., 2018), and miR-423-5p was up-regulated in the circulating blood (Timofeeva et al., 2018), whereas miR-544 and miR-3942 were down-regulated in the placenta tissues (Lykoudi et al., 2018). In late-onset PE, miR-383 and miR-1183 were up-regulated in the placenta tissues (Lykoudi et al., 2018), whereas miR-23b-5p and miR-99b-5p were down-regulated in the circulating blood (Mavreli et al., 2020). However, the functional significance of these miRNAs in the pathogenesis of PE has not been confirmed and elucidated. Although great efforts have been made to explore the pathogenesis of miRNA in PE, most of the samples obtained are from patients with early-onset PE, and few studies have been devoted to comparing the pathogenesis of miRNA in early- and late-onset PE. Further exploration and clarification of the similarities and differences of non-coding RNA involved in the pathology of early- and late-onset PE can provide new ideas for explaining the pathogenesis of PE.

In summary, from the proliferation, invasion, and migration of trophoblast cells to cell cycle progression, changes in the above-described ncRNA expression above may affect the functions of those cells and eventually lead to PE. The discovery of early diagnostic markers is key to early pregnancy intervention. With the development of sequencing technologies, methods for detecting differentially expressed ncRNAs in the placenta and plasma of PE patients have been rapidly established. As research into the expression and function of ncRNA remains in its infancy, exploring the reasons underlying the differences between early- and late-onset PE, the discovery of additional PE-related or PE-specific ncRNAs, and corresponding target gene investigations will be major directions of future research. These studies will help clarify the pathological mechanism of PE and provide a new target for its early prevention.

## Author Contributions

All authors contributed to the writing and editing of the manuscript and approved the submitted version.

## Conflict of Interest

The authors declare that the research was conducted in the absence of any commercial or financial relationships that could be construed as a potential conflict of interest.

## Abbreviations

3 ′: UTR 3 ′ untranslated region
AT1-AA: agonistic autoantibodies against the angiotensin II type 1 receptor
BHLHE40: basic helix-loop-helix family member e40
CCAT1: Colon cancer-related transcription factor 1
CDK: cyclin D-dependent kinases
ceRNA: competitive endogenous RNA
circRNA: circular RNA
dMSCs: decidual-derived mesenchymal stem cells
EMT: epithelial-mesenchymal transition
FOXO1: forkhead box protein O1
HDAC4: histone deacetylase 4
HIF: hypoxia inducible factors
KCNQ1OT1: potassium voltage-gated channel subfamily Q member 1 overlapping transcript 1
LncRNA: long non-coding RNA
MALAT1: metastasis-associated lung adenocarcinoma transcript1
miRNA: microRNA
MMP: matrix metallo protein
MSCs: mesenchymal stem cells
ncRNA: non-coding RNA
PE: preeclampsia
PGK1: phosphoglycerate kinase 1
PGK1P2, phosphoglycerate kinase 1: pseudogene 2
PI3K: phosphatidylinositol 3-kinase
PIK3R2: phosphoinositide-3-Kinase Regulatory Subunit 2
ROCK1: Rho-associated coiled-coil kinase 1
sEng: soluble endothelin
sFlt-1: soluble fms-like tyrosine kinase 1
siRNA: interfering RNA
SPRY4-IT1: sprouty homolog 4 intronic transcript 1
THSD7A: thrombospondin type-1 domain-containing 7A
TUG1: taurine upregulated gene 1
VEGF: vascular endothelial growth factor.

## References

[B1] Ali L. E. Salih M. M. Elhassan E. M. Mohmmed A. A. Adam I. (2019). Placental growth factor, vascular endothelial growth factor, and hypoxia-inducible factor-1alpha in the placentas of women with pre-eclampsia. *J. Matern. Fetal. Neonatal. Med.* 32 2628–2632. 10.1080/14767058.2018.1443066 29455633

[B2] Anton L. Olarerin-George A. O. Hogenesch J. B. Elovitz M. A. (2015). Placental expression of miR-517a/b and miR-517c contributes to trophoblast dysfunction and preeclampsia. *PLoS One* 10:e122707. 10.1371/journal.pone.0122707 25799546 PMC4370750

[B3] Berkane N. Lefevre G. Hertig A. (2007). Angiogenic factors in preeclampsia: so complex, so simple? *Nephrol. Dial. Trans.* 22 2753–2756. 10.1093/ndt/gfm429 17617654

[B4] Brkic J. Dunk C. O’Brien J. Fu G. Nadeem L. Wang Y. L. (2018). MicroRNA-218-5p promotes endovascular trophoblast differentiation and spiral artery remodeling. *Mol. Ther.* 26 2189–2205. 10.1016/j.ymthe.2018.07.009 30061037 PMC6127878

[B5] Bugge M. Bergstrom B. Eide O. K. Solli H. Kjonstad I. F. Stenvik J. (2017). Surface toll-like receptor 3 expression in metastatic intestinal epithelial cells induces inflammatory cytokine production and promotes invasiveness. *J. Biol. Chem.* 292 15408–15425. 10.1074/jbc.M117.784090 28717003 PMC5602399

[B6] Burton G. J. Redman C. W. Roberts J. M. Moffett A. (2019). Pre-eclampsia: pathophysiology and clinical implications. *BMJ* 366:l2381. 10.1136/bmj.l2381 31307997

[B7] Cao C. Li J. Li J. Liu L. Cheng X. Jia R. (2017). Long non-coding RNA Uc.187 Is upregulated in preeclampsia and modulates proliferation. Apoptosis, and Invasion of HTR-8/SVneo Trophoblast Cells. *J. Cell Biochem.* 118 1462–1470. 10.1002/jcb.25805 27883216

[B8] Chatterjee P. Weaver L. E. Doersch K. M. Kopriva S. E. Chiasson V. L. Allen S. J. (2012). Placental toll-like receptor 3 and toll-like receptor 7/8 activation contributesto preeclampsia in humans and mice. *PLoS One* 7:e41884. 10.1371/journal.pone.0041884 22848646 PMC3407075

[B9] Chen F. R. Zheng L. M. Wu D. C. Gong H. M. Cen H. Chen W. C. (2020). [Regulatory relationship between lncRNA KCNQ1OT1 and miR-146a-3p in preeclampsia]. *Zhonghua Fu Chan Ke Za Zhi* 55 535–543. 10.3760/cma.j.cn112141-20200322-00246 32854478

[B10] Chen H. Meng T. Liu X. Sun M. Tong C. Liu J. (2015). Long non-coding RNA MALAT-1 is downregulated in preeclampsia and regulates proliferation, apoptosis, migration and invasion of JEG-3 trophoblast cells. *Int. J. Clin. Exp. Pathol.* 8 12718–12727. 26722461 PMC4680406

[B11] Chen J. Khalil R. A. (2017). Matrix metalloproteinases in normal pregnancy and preeclampsia. *Prog. Mol. Biol. Transl. Sci.* 148 87–165. 10.1016/bs.pmbts.2017.04.001 28662830 PMC5548443

[B12] Chen L. L. (2016). The biogenesis and emerging roles of circular RNAs. *Nat. Rev. Mol. Cell. Biol.* 17 205–211. 10.1038/nrm.2015.32 26908011

[B13] Chen Q. Sousa J. D. Snowise S. Chamley L. Stone P. (2016). Reduction in the severity of early onset severe preeclampsia during gestation may be associated with changes in endothelial cell activation: a pathological case report. *Hypertens Pregnancy* 35 32–41. 10.3109/10641955.2015.1100309 26852788

[B14] Chen S. Zhao G. Miao H. Tang R. Song Y. Hu Y. (2015). MicroRNA-494 inhibits the growth and angiogenesis-regulating potential of mesenchymal stem cells. *Febs Lett* 589 710–717. 10.1016/j.febslet.2015.01.038 25660325

[B15] Chen Y. Ding H. Wei M. Zha W. Guan S. Liu N. (2020). MSC-Secreted exosomal H19 promotes trophoblast cell invasion and migration by downregulating let-7b and upregulating FOXO1. *Mol. Ther. Nucleic Acids* 19 1237–1249. 10.1016/j.omtn.2019.11.031 32069774 PMC7026285

[B16] Cheng D. Jiang S. Chen J. Li J. Ao L. Zhang Y. (2019b). Upregulated long noncoding RNA Linc00261 in pre-eclampsia and its effect on trophoblast invasion and migration via regulating miR-558/TIMP4 signaling pathway. *J. Cell Biochem.* 120 13243–13253. 10.1002/jcb.28598 30891826

[B17] Cheng D. Jiang S. Chen J. Li J. Ao L. Zhang Y. (2019a). The increased lncRNA MIR503HG in preeclampsia modulated trophoblast cell proliferation, invasion, and migration via regulating matrix metalloproteinases and NF-kappaB signaling. *Dis. Mark.* 2019:4976845. 10.1155/2019/4976845 31467616 PMC6701315

[B18] Chim S. S. Shing T. K. Hung E. C. Leung T. Y. Lau T. K. Chiu R. W. (2008). Detection and characterization of placental microRNAs in maternal plasma. *Clin. Chem.* 54 482–490. 10.1373/clinchem.2007.097972 18218722

[B19] Chou S. D. Murshid A. Eguchi T. Gong J. Calderwood S. K. (2015). HSF1 regulation of beta-catenin in mammary cancer cells through control of HuR/elavL1 expression. *Oncogene* 34 2178–2188. 10.1038/onc.2014.177 24954509 PMC4275421

[B20] Delas M. J. Hannon G. J. (2017). lncRNAs in development and disease: from functions to mechanisms. *Open Biol.* 7:170121. 10.1098/rsob.170121 28747406 PMC5541349

[B21] Deng L. Yang S. B. Xu F. F. Zhang J. H. (2015). Long noncoding RNA CCAT1 promotes hepatocellular carcinoma progression by functioning as let-7 sponge. *J. Exp. Clin. Cancer Res.* 34:18. 10.1186/s13046-015-0136-7 25884472 PMC4339002

[B22] Deng N. Lei D. Huang J. Yang Z. Fan C. Wang S. (2019). Circular RNA expression profiling identifies hsa_circ_0011460 as a novel molecule in severe preeclampsia. *Pregnancy Hypertens* 17 216–225. 10.1016/j.preghy.2019.06.009 31487644

[B23] Ding G. C. Chen M. Wang Y. X. Rui C. Xu W. Ding H. J. (2016). MicroRNA-128a-induced apoptosis in HTR-8/SVneo trophoblast cells contributes to pre-eclampsia. *Biomed. Pharmacother.* 81 63–70. 10.1016/j.biopha.2016.03.040 27261578

[B24] Djuranovic S. Nahvi A. Green R. (2011). A parsimonious model for gene regulation by miRNAs. *Science* 331 550–553. 10.1126/science.1191138 21292970 PMC3955125

[B25] Dong D. Khoong Y. Ko Y. Zhang Y. (2020). microRNA-646 inhibits angiogenesis of endothelial progenitor cells in pre-eclamptic pregnancy by targeting the VEGF-A/HIF-1alpha axis. *Exp. Ther. Med.* 20 1879–1888. 10.3892/etm.2020.8929 32782496 PMC7401288

[B26] Dong X. Zhang Y. Chen X. Xue M. (2020). Long noncoding RNA LINC00511 regulates the proliferation, apoptosis, invasion and autophagy of trophoblast cells to mediate pre-eclampsia progression through modulating the miR-31-5p/homeobox protein A7 axis. *J. Obstet. Gynaecol. Res.* 46 1298–1309. 10.1111/jog.14344 32558037

[B27] Duhig K. Vandermolen B. Shennan A. (2018). Recent advances in the diagnosis and management of pre-eclampsia. *F1000 Res.* 7:242. 10.12688/f1000research.12249.1 29560262 PMC5832913

[B28] Eghbal-Fard S. Yousefi M. Heydarlou H. Ahmadi M. Taghavi S. Movasaghpour A. (2019). The imbalance of Th17/Treg axis involved in the pathogenesis of preeclampsia. *J. Cell. Physiol.* 234 5106–5116. 10.1002/jcp.27315 30277561

[B29] Faas M. M. De Vos P. (2018). Innate immune cells in the placental bed in healthy pregnancy and preeclampsia. *Placenta* 69 125–133. 10.1016/j.placenta.2018.04.012 29748088

[B30] Fan M. Xu Y. Hong F. Gao X. Xin G. Hong H. (2016). Rac1/beta-catenin signalling pathway contributes to trophoblast cell invasion bytargeting snail and MMP9. *Cell Physiol. Biochem.* 38 1319–1332. 10.1159/000443076 27008403

[B31] Fang M. Du H. Han B. Xia G. Shi X. Zhang F. (2017). Hypoxia-inducible microRNA-218 inhibits trophoblast invasion by targeting LASP1:Implications for preeclampsia development. *Int. J. Biochem. Cell. Biol.* 87 95–103. 10.1016/j.biocel.2017.04.005 28412444

[B32] Fu G. Ye G. Nadeem L. Ji L. Manchanda T. Wang Y. (2013). MicroRNA-376c impairs transforming growth factor-beta and nodal signaling to promote trophoblast cell proliferation and invasion. *Hypertension* 61 864–872. 10.1161/HYPERTENSIONAHA.111.203489 23424236

[B33] Fu J. Y. Xiao Y. P. Ren C. L. Guo Y. W. Qu D. H. Zhang J. H. (2018). Up-regulation of miR-517-5p inhibits ERK/MMP-2 pathway: potential role in preeclampsia. *Eur. Rev. Med. Pharmacol. Sci.* 22 6599–6608. 10.26355/eurrev_201810_16134 30402831

[B34] Gan L. Liu Z. Wei M. Chen Y. Yang X. Chen L. (2017). MiR-210 and miR-155 as potential diagnostic markers for pre-eclampsia pregnancies. *Medicine (Baltimore)* 96:e7515. 10.1097/MD.0000000000007515 28700503 PMC5515775

[B35] Gao Y. Guo X. Li Y. Sha W. She R. (2019). The decreased lncRNA ZEB2-AS1 in pre-eclampsia controls the trophoblastic cell line HTR-8/SVneo’s invasive and migratory abilities via the miR-149/PGF axis. *J. Cell. Biochem.* 120 17677–17686. 10.1002/jcb.29034 31148230

[B36] Gao Y. She R. Wang Q. Li Y. Zhang H. (2018). Up-regulation of miR-299 suppressed the invasion and migration of HTR-8/SVneo trophoblast cells partly via targeting HDAC2 in pre-eclampsia. *Biomed. Pharmacother.* 97 1222–1228. 10.1016/j.biopha.2017.11.053 29145147

[B37] Geldenhuys J. Rossouw T. M. Lombaard H. A. Ehlers M. M. Kock M. M. (2018). Disruption in the regulation of immune responses in the placental subtype of preeclampsia. *Front. Immunol.* 9:1659. 10.3389/fimmu.2018.01659 30079067 PMC6062603

[B38] Gomes A. Q. Nolasco S. Soares H. (2013). Non-coding RNAs: multi-tasking molecules in the cell. *Int. J. Mol. Sci.* 14 16010–16039. 10.3390/ijms140816010 23912238 PMC3759897

[B39] Gong F. Chai W. Wang J. Cheng H. Shi Y. Cui L. (2020a). miR-214-5p suppresses the proliferation, migration and invasion of trophoblast cells in pre-eclampsia by targeting jagged 1 to inhibit notch signaling pathway. *Acta Histochem* 122:151527. 10.1016/j.acthis.2020.151527 32113857

[B40] Gong F. Cheng H. Shi Y. Cui L. Jia G. (2020b). LncRNA TDRG1/miR-214-5p axis affects preeclampsia by modulating trophoblast cells. *Cell. Biochem. Funct.* 38 352–361. 10.1002/cbf.3480 31885100

[B41] Gude N. M. Roberts C. T. Kalionis B. King R. G. (2004). Growth and function of the normal human placenta. *Thromb. Res.* 114 397–407. 10.1016/j.thromres.2004.06.038 15507270

[B42] Guo L. Zhao Y. Yang S. Zhang H. Chen F. (2014). An integrated analysis of miRNA, lncRNA, and mRNA expression profiles. *Biomed. Res. Int.* 2014:345605. 10.1155/2014/345605 25045664 PMC4086520

[B43] Gusar V. Timofeeva A. Chagovets V. Kan N. Vasilchenko O. Prozorovskaya K. (2020). Preeclampsia: the interplay between oxygen-sensitive miRNAs and erythropoietin. *J. Clin. Med.* 9:574. 10.3390/jcm9020574 32093169 PMC7073952

[B44] Guttman M. Rinn J. L. (2012). Modular regulatory principles of large non-coding RNAs. *Nature* 482 339–346. 10.1038/nature10887 22337053 PMC4197003

[B45] Han B. Chao J. Yao H. (2018). Circular RNA and its mechanisms in disease: From the bench to the clinic. *Pharmacol. Ther.* 187 31–44. 10.1016/j.pharmthera.2018.01.010 29406246

[B46] Han X. Niu C. Zuo Z. Wang Y. Yao L. Sun L. (2020). MiR-342-3p inhibition promotes cell proliferation and invasion by directly targeting ID4 in pre-eclampsia. *J. Obstet. Gynaecol. Res.* 46 49–57. 10.1111/jog.14150 31749272

[B47] Hansen T. B. Jensen T. I. Clausen B. H. Bramsen J. B. Finsen B. Damgaard C. K. (2013). Natural RNA circles function as efficient microRNA sponges. *Nature* 495 384–388. 10.1038/nature11993 23446346

[B48] Hong F. Li Y. Xu Y. (2014). Decreased placental miR-126 expression and vascular endothelial growth factor levels in patients with pre-eclampsia. *J. Int. Med. Res.* 42 1243–1251. 10.1177/0300060514540627 25341970

[B49] Hromadnikova I. Dvorakova L. Kotlabova K. Krofta L. (2019). The prediction of gestational hypertension, preeclampsia and fetal growth restriction via the first trimester screening of plasma exosomal C19MC microRNAs. *Int. J. Mol. Sci.* 20:2972. 10.3390/ijms20122972 31216670 PMC6627682

[B50] Hromadnikova I. Kotlabova K. Ivankova K. Vedmetskaya Y. Krofta L. (2017). Profiling of cardiovascular and cerebrovascular disease associated microRNA expression in umbilical cord blood in gestational hypertension, preeclampsia andfetal growth restriction. *Int. J. Cardiol.* 249 402–409. 10.1016/j.ijcard.2017.07.045 29121743

[B51] Hromadnikova I. Kotlabova K. Ondrackova M. Kestlerova A. Novotna V. Hympanova L. (2013). Circulating C19MC microRNAs in preeclampsia, gestational hypertension, and fetalgrowth restriction. *Med. Inf.* 2013:186041. 10.1155/2013/186041 24347821 PMC3848305

[B52] Hu X. Ao J. Li X. Zhang H. Wu J. Cheng W. (2018). Competing endogenous RNA expression profiling in pre-eclampsia identifies hsa_circ_0036877 as a potential novel blood biomarker for early pre-eclampsia. *Clin. Epigenetics* 10:48. 10.1186/s13148-018-0482-3 29643944 PMC5891938

[B53] Hu X. Li X. Tian G. G. Zhang H. Cheng W. Wu J. (2019). Expression profiling dataset of competing endogenous RNA in pre-eclampsia. *Data Brief.* 27:104795. 10.1016/j.dib.2019.104795 31799347 PMC6881654

[B54] Huang X. Wu L. Zhang G. Tang R. Zhou X. (2019). Elevated microrna-181a-5p contributes to trophoblast dysfunction and preeclampsia. *Reprod. Sci.* 26 1121–1129. 10.1177/1933719118808916 30376765

[B55] Huppertz B. Kadyrov M. Kingdom J. C. (2006). Apoptosis and its role in the trophoblast. *Am. J. Obstet. Gynecol.* 195 29–39. 10.1016/j.ajog.2005.07.039 16579915

[B56] Ishibashi O. Ohkuchi A. Ali M. M. Kurashina R. Luo S. S. Ishikawa T. (2012). Hydroxysteroid (17-beta) dehydrogenase 1 is dysregulated by miR-210 and miR-518cthat are aberrantly expressed in preeclamptic placentas: a novel marker for predicting preeclampsia. *Hypertension* 59 265–273. 10.1161/HYPERTENSIONAHA.111.180232 22203747

[B57] Ji L. Zhang L. Li Y. Guo L. Cao N. Bai Z. (2017). MiR-136 contributes to pre-eclampsia through its effects on apoptosis and angiogenesis of mesenchymal stem cells. *Placenta* 50 102–109. 10.1016/j.placenta.2017.01.102 28161054

[B58] Ji P. Diederichs S. Wang W. Boing S. Metzger R. Schneider P. M. (2003). MALAT-1, a novel noncoding RNA, and thymosin beta4 predict metastasis and survival in early-stage non-small cell lung cancer. *Oncogene* 22 8031–8041. 10.1038/sj.onc.1206928 12970751

[B59] Jiang F. Li J. Wu G. Miao Z. Lu L. Ren G. (2015). Upregulation of microRNA335 and microRNA584 contributes to the pathogenesis of severe preeclampsia through downregulation of endothelial nitric oxide synthase. *Mol. Med. Rep.* 12 5383–5390. 10.3892/mmr.2015.4018 26133786

[B60] Jiang L. Long A. Tan L. Hong M. Wu J. Cai L. (2017). Elevated microRNA-520g in pre-eclampsia inhibits migration and invasion of trophoblasts. *Placenta* 51 70–75. 10.1016/j.placenta.2017.02.001 28292471

[B61] Jiang M. Lash G. E. Zhao X. Long Y. Guo C. Yang H. (2018). CircRNA-0004904, CircRNA-0001855, and papp-a: potential novel biomarkers for theprediction of preeclampsia. *Cell Physiol. Biochem.* 46 2576–2586. 10.1159/000489685 29758559

[B62] Jin M. Li H. Xu H. Huo G. Yao Y. (2017). MicroRNA-20b inhibits trophoblast cell migration and invasion by targeting MMP-2. *Int. J. Clin. Exp. Pathol.* 10 10901–10909. 31966433 PMC6965854

[B63] Kim J. Lee K. S. Kim J. H. Lee D. K. Park M. Choi S. (2017). Aspirin prevents TNF-alpha-induced endothelial cell dysfunction by regulating the NF-kappaB-dependent miR-155/eNOS pathway: role of a miR-155/eNOS axis in preeclampsia. *Free Radic Biol. Med.* 104 185–198. 10.1016/j.freeradbiomed.2017.01.010 28087411

[B64] Kopriva S. E. Chiasson V. L. Mitchell B. M. Chatterjee P. (2013). TLR3-induced placental miR-210 down-regulates the STAT6/interleukin-4 pathway. *PLoS One* 8:e67760. 10.1371/journal.pone.0067760 23844087 PMC3699491

[B65] Lasda E. Parker R. (2014). Circular RNAs: diversity of form and function. *RNA* 20 1829–1842. 10.1261/rna.047126.114 25404635 PMC4238349

[B66] Lavallee L. (2015). Clinical presentation, assessment and management of pre-eclampsia. *Nurs. Stand.* 29 51–59. 10.7748/ns.29.45.51.e9952 26153971

[B67] Li J. Wang J. M. Liu Y. H. Zhang Z. Han N. Wang J. Y. (2017c). [Effect of microRNA-106b on the invasion and proliferation of trophoblasts through targeting MMP-2]. *Zhonghua Fu Chan Ke Za Zhi* 52 327–332. 10.3760/cma.j.issn.0529-567X.2017.05.007 28545271

[B68] Li J. Wu G. Cao Y. Hou Z. (2019). Roles of miR-210 in the pathogenesis of pre-eclampsia. *Arch. Med. Sci.* 15 183–190. 10.5114/aoms.2018.73129 30697269 PMC6348360

[B69] Li L. Zhang X. Hong S. L. Chen Y. Ren G. H. (2018). Long non-coding HOTTIP regulates preeclampsia by inhibiting RND3. *Eur. Rev. Med. Pharmacol. Sci.* 22 3277–3285. 10.26355/eurrev_201806_15146 29917176

[B70] Li P. Guo W. Du L. Zhao J. Wang Y. Liu L. (2013). microRNA-29b contributes to pre-eclampsia through its effects on apoptosis, invasion and angiogenesis of trophoblast cells. *Clin. Sci. (Lond)* 124 27–40. 10.1042/CS20120121 22716646

[B71] Li Q. Wang T. Huang S. Zuo Q. Jiang Z. Yang N. (2020). LncRNA MALAT1 affects the migration and invasion of trophoblast cells by regulating FOS expression in early-onset preeclampsia. *Pregnancy Hypertens* 21 50–57. 10.1016/j.preghy.2020.05.001 32408074

[B72] Li Q. Zhang J. Su D. M. Guan L. N. Mu W. H. Yu M. (2019). lncRNA TUG1 modulates proliferation, apoptosis, invasion, and angiogenesis via targeting miR-29b in trophoblast cells. *Hum. Geno.* 13:50. 10.1186/s40246-019-0237-z 31519209 PMC6743181

[B73] Li X. Li C. Dong X. Gou W. (2014). MicroRNA-155 inhibits migration of trophoblast cells and contributes to the pathogenesis of severe preeclampsia by regulating endothelial nitric oxide synthase. *Mol. Med. Rep.* 10 550–554. 10.3892/mmr.2014.2214 24806148

[B74] Li X. Song Y. Liu D. Zhao J. Xu J. Ren J. (2017b). MiR-495 Promotes senescence of mesenchymal stem cells by targeting bmi-1. *Cell. Physiol. Biochem.* 42 780–796. 10.1159/000478069 28628915

[B75] Li X. Song Y. Liu F. Liu D. Miao H. Ren J. (2017a). Long non-coding RNA MALAT1 promotes proliferation. angiogenesis, and immunosuppressive properties of mesenchymal stem cells by inducing VEGF and IDO. *J. Cell. Biochem.* 118 2780–2791. 10.1002/jcb.25927 28176360

[B76] Li Z. Ruan Y. Zhang H. Shen Y. Li T. Xiao B. (2019c). Tumor-suppressive circular RNAs: mechanisms underlying their suppression of tumor occurrence and use as therapeutic targets. *Cancer Sci.* 110 3630–3638. 10.1111/cas.14211 31599076 PMC6890437

[B77] Ling Z. Chen M. Li T. Qian Y. Li C. (2021). MiR-141-3p downregulation promotes tube formation, migration, invasion and inhibits apoptosis in hypoxia-induced human umbilical vein endothelial cells by targeting Notch2. *Reprod. Biol.* 21:100483. 10.1016/j.repbio.2021.100483 33631423

[B78] Lisonkova S. Sabr Y. Mayer C. Young C. Skoll A. Joseph K. S. (2014). Maternal morbidity associated with early-onset and late-onset preeclampsia. *Obstet Gynecol.* 124 771–781. 10.1097/AOG.0000000000000472 25198279

[B79] Liu F. Wu K. Wu W. Chen Y. Wu H. Wang H. (2018). miR203 contributes to preeclampsia via inhibition of VEGFA expression. *Mol. Med. Rep.* 17 5627–5634. 10.3892/mmr.2018.8558 29436641 PMC5866003

[B80] Liu L. Wang Y. Fan H. Zhao X. Liu D. Hu Y. (2012). MicroRNA-181a regulates local immune balance by inhibiting proliferation and immunosuppressive properties of mesenchymal stem cells. *Stem. Cells* 30 1756–1770. 10.1002/stem.1156 22714950

[B81] Liu S. Xie X. Lei H. Zou B. Xie L. (2019). Identification of key circRNAs/lncRNAs/miRNAs/mRNAs and pathways in preeclampsiausing bioinformatics analysis. *Med. Sci. Monit.* 25 1679–1693. 10.12659/MSM.912801 30833538 PMC6413561

[B82] Liu W. Liu X. Luo M. Liu X. Luo Q. Tao H. (2017). dNK derived IFN-gamma mediates VSMC migration and apoptosis via the induction ofLncRNA MEG3: A role in uterovascular transformation. *Placenta* 50 32–39. 10.1016/j.placenta.2016.12.023 28161059

[B83] Liu X. Chen H. Kong W. Zhang Y. Cao L. Gao L. (2017). Down-regulated long non-coding RNA-ATB in preeclampsia and its effect on suppressing migration, proliferation, and tube formation of trophoblast cells. *Placenta* 49 80–87. 10.1016/j.placenta.2016.12.004 28012459

[B84] Lockwood C. J. Yen C. F. Basar M. Kayisli U. A. Martel M. Buhimschi I. (2008). Preeclampsia-related inflammatory cytokines regulate interleukin-6 expression inhuman decidual cells. *Am. J. Pathol.* 172 1571–1579. 10.2353/ajpath.2008.070629 18467705 PMC2408417

[B85] Long W. Rui C. Song X. Dai X. Xue X. Lu Y. (2016). Distinct expression profiles of lncRNAs between early-onset preeclampsia and preterm controls. *Clin. Chim. Acta* 463 193–199. 10.1016/j.cca.2016.10.036 27816668

[B86] Lu W. Ma Y. Y. Shao Q. Q. Liang J. Qi T. T. Huang Y. (2020). ROS/p53/miR3355p/Sp1 axis modulates the migration and epithelial to mesenchymal transition of JEG3 cells. *Mol. Med. Rep.* 21 1208–1216. 10.3892/mmr.2019.10901 31894323 PMC7003020

[B87] Lukiw W. J. (2013). Circular RNA (circRNA) in Alzheimer’s disease (AD). *Front. Genet.* 4:307. 10.3389/fgene.2013.00307 24427167 PMC3875874

[B88] Luo L. Ye G. Nadeem L. Fu G. Yang B. B. Honarparvar E. (2012). MicroRNA-378a-5p promotes trophoblast cell survival, migration and invasion by targeting Nodal. *J. Cell Sci.* 125(Pt. 13) 3124–3132. 22454525 10.1242/jcs.096412

[B89] Luo R. Shao X. Xu P. Liu Y. Wang Y. Zhao Y. (2014). MicroRNA-210 contributes to preeclampsia by downregulating potassium channel modulatory factor 1. *Hypertension* 64 839–845. 10.1161/HYPERTENSIONAHA.114.03530 24980667

[B90] Luo R. Wang Y. Xu P. Cao G. Zhao Y. Shao X. (2016). Hypoxia-inducible miR-210 contributes to preeclampsia via targeting thrombospondin type I domain containing 7A. *Sci Rep* 6 19588. 10.1038/srep19588 26796133 PMC4726282

[B91] Lv Y. Lu X. Li C. Fan Y. Ji X. Long W. (2019). miR-145-5p promotes trophoblast cell growth and invasion by targeting FLT1. *Life Sci.* 239:117008. 10.1016/j.lfs.2019.117008 31669240

[B92] Lykoudi A. Kolialexi A. Lambrou G. I. Braoudaki M. Siristatidis C. Papaioanou G. K. (2018). Dysregulated placental microRNAs in early and Late onset preeclampsia. *Placenta* 61 24–32. 10.1016/j.placenta.2017.11.005 29277268

[B93] Ma Y. Liang X. Wu H. Zhang C. Ma Y. (2019). Long noncoding RNA NR_002794 is upregulated in preeclampsia and regulates the proliferation, apoptosis and invasion of trophoblast cells. *Mol. Med. Rep.* 20 4567–4575. 10.3892/mmr.2019.10701 31702023 PMC6797946

[B94] Mavreli D. Lykoudi A. Lambrou G. Papaioannou G. Vrachnis N. Kalantaridou S. (2020). Deep sequencing identified dysregulated circulating microRNAs in late onset preeclampsia. *In. Vivo.* 34 2317–2324. 10.21873/invivo.12044 32871756 PMC7652460

[B95] McLaughlin K. Zhang J. Lye S. J. Parker J. D. Kingdom J. C. (2018). Phenotypes of Pregnant Women Who Subsequently Develop Hypertension in Pregnancy. *J. Am. Heart Assoc.* 7:14. 10.1161/JAHA.118.009595 30007936 PMC6064839

[B96] Memczak S. Jens M. Elefsinioti A. Torti F. Krueger J. Rybak A. (2013). Circular RNAs are a large class of animal RNAs with regulatory potency. *Nature* 495 333–338. 10.1038/nature11928 23446348

[B97] Motawi T. Sabry D. Maurice N. W. Rizk S. M. (2018). Role of mesenchymal stem cells exosomes derived microRNAs; miR-136, miR-494 and miR-495 in pre-eclampsia diagnosis and evaluation. *Arch. Biochem. Biophys.* 659 13–21. 10.1016/j.abb.2018.09.023 30261165

[B98] Mouillet J. F. Chu T. Nelson D. M. Mishima T. Sadovsky Y. (2010). MiR-205 silences MED1 in hypoxic primary human trophoblasts. *Faseb. J.* 24 2030–2039. 10.1096/fj.09-149724 20065103 PMC2874470

[B99] Myatt L. (2010). Review: reactive oxygen and nitrogen species and functional adaptation of the placenta. *Placenta* 31(Suppl.) S66–S69. 10.1016/j.placenta.2009.12.021 20110125 PMC2832707

[B100] Ou Y. Zhu L. Wei X. Bai S. Chen M. Chen H. (2020). Circular RNA circ_0111277 attenuates human trophoblast cell invasion and migration by regulating miR-494/HTRA1/Notch-1 signal pathway in pre-eclampsia. *Cell Death Dis.* 11:479. 10.1038/s41419-020-2679-6 32587240 PMC7316814

[B101] Oudejans C. B. Poutsma A. Michel O. J. Thulluru H. K. Mulders J. van de Vrugt H. J. (2016). Noncoding RNA-regulated gain-of-function of STOX2 in finnish pre-eclamptic families. *Sci. Rep.* 6:32129. 10.1038/srep32129 27555360 PMC4995371

[B102] Pamudurti N. R. Bartok O. Jens M. Ashwal-Fluss R. Stottmeister C. Ruhe L. (2017). Translation of CircRNAs. *Mol. Cell.* 66 9–21. 10.1016/j.molcel.2017.02.021 28344080 PMC5387669

[B103] Salomon C. Guanzon D. Scholz-Romero K. Longo S. Correa P. Illanes S. E. (2017). Placental exosomes as early biomarker of preeclampsia: potential role of exosomal microRNAs across gestation. *J. Clin. Endocrinol. Metab.* 102 3182–3194. 10.1210/jc.2017-00672 28531338

[B104] Schjenken J. E. Zhang B. Chan H. Y. Sharkey D. J. Fullston T. Robertson S. A. (2016). miRNA regulation of immune tolerance in early pregnancy. *Am. J. Reprod. Immunol.* 75 272–280. 10.1111/aji.12490 26804209

[B105] Seifeddine R. Dreiem A. Blanc E. Fulchignoni-Lataud M. C. Le Frere B. M. Lecuru F. (2008). Hypoxia down-regulates CCAAT/enhancer binding protein-alpha expression in breastcancer cells. *Cancer Res.* 68 2158–2165. 10.1158/0008-5472.CAN-07-1190 18381421

[B106] Seyom E. Abera M. Tesfaye M. Fentahun N. (2015). Maternal and fetal outcome of pregnancy related hypertension in Mettu Karl Referral Hospital. *Ethiopia. J. Ovarian Res.* 8:10. 10.1186/s13048-015-0135-5 25824330 PMC4374296

[B107] Sheikh A. M. Small H. Y. Currie G. Delles C. (2016). Systematic review of micro-rna expression in pre-eclampsia identifies a number of common pathways associated with the disease. *PLoS. One* 11:e160808. 10.1371/journal.pone.0160808 27529341 PMC4986940

[B108] Shen X. Y. Zheng L. L. Huang J. Kong H. F. Chang Y. J. Wang F. (2019). CircTRNC18 inhibits trophoblast cell migration and epithelial-mesenchymal transition by regulating miR-762/Grhl2 pathway in pre-eclampsia. *RNA Biol.* 16 1565–1573. 10.1080/15476286.2019.1644591 31354028 PMC6779405

[B109] Shi L. Fisslthaler B. Zippel N. Fromel T. Hu J. Elgheznawy A. (2013). MicroRNA-223 antagonizes angiogenesis by targeting beta1 integrin and preventinggrowth factor signaling in endothelial cells. *Circ. Res.* 113 1320–1330. 10.1161/CIRCRESAHA.113.301824 24044949

[B110] Shi Z. She K. Li H. Yuan X. Han X. Wang Y. (2019). MicroRNA-454 contributes to sustaining the proliferation and invasion of trophoblast cells through inhibiting Nodal/ALK7 signaling in pre-eclampsia. *Chem. Biol. Interact.* 298 8–14. 10.1016/j.cbi.2018.10.012 30367833

[B111] Sohlberg S. Mulic-Lutvica A. Lindgren P. Ortiz-Nieto F. Wikstrom A. K. Wikstrom J. (2014). Placental perfusion in normal pregnancy and early and late preeclampsia: a magnetic resonance imaging study. *Placenta* 35 202–206. 10.1016/j.placenta.2014.01.008 24529946

[B112] Song J. Li Y. An R. F. (2015). Identification of early-onset preeclampsia-related genes and microRNAs by bioinformatics approaches. *Rep. Sci.* 22 954–963. 10.1177/1933719115570898 25717061

[B113] Song X. Rui C. Meng L. Zhang R. Shen R. Ding H. (2017). Long non-coding RNA RPAIN regulates the invasion and apoptosis of trophoblast cell lines via complement protein C1q. *Oncotarget* 8 7637–7646. 10.18632/oncotarget.13826 28032589 PMC5352349

[B114] Sonkar R. Kay M. K. Choudhury M. P. F. O. S. (2019). Modulates interactive epigenetic regulation in first-trimester human trophoblast cell line HTR-8/SVneo. *Chem. Res. Toxicol.* 32 2016–2027. 10.1021/acs.chemrestox.9b00198 31508952

[B115] Souza J. P. Gulmezoglu A. M. Vogel J. Carroli G. Lumbiganon P. Qureshi Z. (2013). Moving beyond essential interventions for reduction of maternal mortality (the WHO Multicountry Survey on Maternal and Newborn Health): a cross-sectional study. *Lancet* 381 1747–1755. 10.1016/S0140-6736(13)60686-8 23683641

[B116] Steegers E. A. von Dadelszen P. Duvekot J. J. Pijnenborg R. (2010). Pre-eclampsia. *Lancet* 376 631–644. 10.1016/S0140-6736(10)60279-6 20598363

[B117] Stubert J. Koczan D. Richter D. U. Dieterich M. Ziems B. Thiesen H. J. (2014). miRNA expression profiles determined in maternal sera of patients with HELLP syndrome. *Hypertens Pregnancy* 33 215–235. 10.3109/10641955.2013.858743 24304191

[B118] Sun K. Lai E. C. (2013). Adult-specific functions of animal microRNAs. *Nat. Rev. Genet.* 14 535–548. 10.1038/nrg3471 23817310 PMC4136762

[B119] Sun M. Kraus W. L. (2015). From discovery to function: the expanding roles of long noncoding RNAs in physiology and disease. *Endocr. Rev.* 36 25–64. 10.1210/er.2014-1034 25426780 PMC4309736

[B120] Tian J. Liu Y. Hu M. Zheng Y. Xu P. Zhang L. (2020). Upregulated LncZBTB39 in pre-eclampsia and its effects on trophoblast invasion and migration via antagonizing the inhibition of miR-210 on THSD7A expression. *Eur. J. Obstet. Gynecol. Rep. Biol.* 248 164–171. 10.1016/j.ejogrb.2020.03.035 32222649

[B121] Timofeeva A. V. Gusar V. A. Kan N. E. Prozorovskaya K. N. Karapetyan A. O. Bayev O. R. (2018). Identification of potential early biomarkers of preeclampsia. *Placenta* 61 61–71. 10.1016/j.placenta.2017.11.011 29277273

[B122] Tong J. Yang J. Lv H. Lv S. Zhang C. Chen Z. J. (2018). Dysfunction of pseudogene PGK1P2 is involved in preeclampsia by acting as a competing endogenous RNA of PGK1. *Pregnancy Hypertens* 13 37–45. 10.1016/j.preghy.2018.05.003 30177069

[B123] Tsai P. Y. Li S. H. Chen W. N. Tsai H. L. Su M. T. (2017). Differential miR-346 and miR-582-3p expression in association with selected maternal and fetal complications. *Int. J. Mol. Sci.* 18:1570. 10.3390/ijms18071570 28753968 PMC5536058

[B124] Wang C. Y. Tsai P. Y. Chen T. Y. Tsai H. L. Kuo P. L. Su M. T. (2019). Elevated miR-200a and miR-141 inhibit endocrine gland-derived vascular endothelial growth factor expression and ciliogenesis in preeclampsia. *J. Physiol.* 597 3069–3083. 10.1113/JP277704 31026335

[B125] Wang H. Zhang L. Guo X. Bai Y. Li Y. X. Sha J., et al. (2018). MiR-195 modulates oxidative stress-induced apoptosis and mitochondrial energy production in human trophoblasts via flavin adenine dinucleotide-dependent oxidoreductase domain-containing protein 1 and pyruvate dehydrogenase phosphatase regulatory subunit. *J. Hypertens* 36 306–318. 10.1097/HJH.0000000000001529 28858979

[B126] Wang P. Xue Y. Han Y. Lin L. Wu C. Xu S. (2014). The STAT3-binding long noncoding RNA lnc-DC controls human dendritic cell differentiation. *Science* 344 310–313. 10.1126/science.1251456 24744378

[B127] Wang Q. Lu X. Li C. Zhang W. Lv Y. Wang L. (2019). Down-regulated long non-coding RNA PVT1 contributes to gestational diabetes mellitus and preeclampsia via regulation of human trophoblast cells. *Biomed. Pharmacother.* 120:109501. 10.1016/j.biopha.2019.109501 31627090

[B128] Wang R. Liu W. Liu X. Liu X. Tao H. Wu D. (2019). MicroRNA-210 regulates human trophoblast cell line HTR-8/SVneo function by attenuating Notch1 expression: Implications for the role of microRNA-210 in pre-eclampsia. *Mol Reprod Dev* 86 896–907. 10.1002/mrd.23154 31115130

[B129] Wang W. T. Ye H. Wei P. P. Han B. W. He B. Chen Z. H. (2016). LncRNAs H19 and HULC, activated by oxidative stress, promote cell migration and invasion in cholangiocarcinoma through a ceRNA manner. *J. Hematol. Oncol.* 9:117. 10.1186/s13045-016-0348-0 27809873 PMC5093965

[B130] Wang W. Feng L. Zhang H. Hachy S. Satohisa S. Laurent L. C. (2012). Preeclampsia up-regulates angiogenesis-associated microRNA (i.e., miR-17, -20a, and -20b) that target ephrin-B2 and EPHB4 in human placenta. *J. Clin. Endocrinol. Metab* 97 E1051–E1059. 10.1210/jc.2011-3131 22438230 PMC3387422

[B131] Wang X. Q. Zhou W. J. Hou X. X. Fu Q. Li D. J. (2018). Trophoblast-derived CXCL16 induces M2 macrophage polarization that in turn inactivates NK cells at the maternal-fetal interface. *Cell Mol. Immunol.* 15 1038–1046. 10.1038/s41423-018-0019-x 29588487 PMC6269500

[B132] Wang Y. Cheng K. Zhou W. Liu H. Yang T. Hou P. (2019). miR-141-5p regulate ATF2 via effecting MAPK1/ERK2 signaling to promote preeclampsia. *Biomed. Pharmacother.* 115:108953. 10.1016/j.biopha.2019.108953 31075732

[B133] Wang Y. Dong Q. Gu Y. Groome L. J. (2016). Up-regulation of miR-203 expression induces endothelial inflammatory response: potential role in preeclampsia. *Am. J. Reprod. Immunol.* 76 482–490. 10.1111/aji.12589 27753461 PMC5118090

[B134] Wang Y. Zhang Y. Wang H. Wang J. Zhang Y. Wang Y. (2014). Aberrantly up-regulated miR-20a in pre-eclampsic placenta compromised the proliferative and invasive behaviors of trophoblast cells by targeting forkhead box protein A1. *Int J Biol Sci* 10 973–982. 10.7150/ijbs.9088 25210495 PMC4159688

[B135] Weber M. Gohner C. San M. S. Vattai A. Hutter S. Parraga M. (2016). Unique trophoblast stem cell- and pluripotency marker staining patterns depending on gestational age and placenta-associated pregnancy complications. *Cell Adh. Migr.* 10 56–65. 10.1080/19336918.2016.1142035 26914354 PMC4853049

[B136] Wen Z. Chen Y. Long Y. Yu J. Li M. (2018). Tumor necrosis factor-alpha suppresses the invasion of HTR-8/SVneo trophoblast cells through microRNA-145-5p-mediated downregulation of Cyr61. *Life Sci.* 209 132–139. 10.1016/j.lfs.2018.08.005 30081007

[B137] Wu D. Chen X. Wang L. Chen F. Cen H. Shi L. (2019). Hypoxia-induced microRNA-141 regulates trophoblast apoptosis, invasion, and vascularization by blocking CXCL12beta/CXCR2/4 signal transduction. *Biomed. Pharmacother.* 116:108836. 10.1016/j.biopha.2019.108836 31004838

[B138] Wu H. Y. Wang X. H. Liu K. Zhang J. L. (2020). LncRNA MALAT1 regulates trophoblast cells migration and invasion via miR-206/IGF-1 axis. *Cell Cycle* 19 39–52. 10.1080/15384101.2019.1691787 31774373 PMC6927728

[B139] Wu J. L. Wang Y. G. Gao G. M. Feng L. Guo N. Zhang C. X. (2019). Overexpression of lncRNA TCL6 promotes preeclampsia progression by regulating PTEN. *Eur. Rev. Med. Pharmacol. Sci.* 23 4066–4072. 10.26355/eurrev_201905_17907 31173275

[B140] Wu L. Song W. Y. Xie Y. Hu L. L. Hou X. M. Wang R. (2018). miR-181a-5p suppresses invasion and migration of HTR-8/SVneo cells by directly targeting IGF2BP2. *Cell Death Dis.* 9:16. 10.1038/s41419-017-0045-0 29339719 PMC5833820

[B141] Wu L. Zhou H. Lin H. Qi J. Zhu C. Gao Z. (2012). Circulating microRNAs are elevated in plasma from severe preeclamptic pregnancies. *Reproduction* 143 389–397. 10.1530/REP-11-0304 22187671

[B142] Wu P. van den Berg C. Alfirevic Z. O’Brien S. Rothlisberger M. Baker P. N. (2015). Early pregnancy biomarkers in pre-eclampsia: a systematic review and meta-analysis. *Int. J. Mol. Sci.* 16 23035–23056. 10.3390/ijms160923035 26404264 PMC4613350

[B143] Xia S. Feng J. Lei L. Hu J. Xia L. Wang J. (2017). Comprehensive characterization of tissue-specific circular RNAs in the human andmouse genomes. *Brief. Bioinform.* 18 984–992. 10.1093/bib/bbw081 27543790

[B144] Xin G. Du J. Liu M. Y. Xu Y. P. (2017). Upregulation of MiR-29b contributes to mesenchymal stem cell dysfunction in patients with severe pre-eclampsia. *Int. J. Clin. Exp. Pathol.* 10 10243–10251. 31966358 PMC6965791

[B145] Xu J. Xia Y. Zhang H. Guo H. Feng K. Zhang C. (2018). Overexpression of long non-coding RNA H19 promotes invasion and autophagy via the PI3K/AKT/mTOR pathways in trophoblast cells. *Biomed. Pharmacother.* 101 691–697. 10.1016/j.biopha.2018.02.134 29522949

[B146] Xu Y. Lian Y. Zhang Y. Huang S. Zuo Q. Yang N. (2018). The long non-coding RNA PVT1 represses ANGPTL4 transcription through binding with EZH2 in trophoblast cell. *J. Cell Mol. Med.* 22 1272–1282. 10.1111/jcmm.13405 29193797 PMC5783862

[B147] Xu Y. Xia X. Jiang Y. Wu D. Wang S. Fu S. (2020). Down-regulated lncRNA AGAP2-AS1 contributes to pre-eclampsia as a competing endogenous RNA for JDP2 by impairing trophoblastic phenotype. *J. Cell. Mol. Med.* 24 4557–4568. 10.1111/jcmm.15113 32150333 PMC7176850

[B148] Xue P. Zheng M. Diao Z. Shen L. Liu M. Gong P. (2013). miR-155^∗^ mediates suppressive effect of PTEN 3’-untranslated region on AP-1/NF-kappaB pathway in HTR-8/SVneo cells. *Placenta* 34 650–656. 10.1016/j.placenta.2013.04.015 23684381

[B149] Yan T. Liu Y. Cui K. Hu B. Wang F. Zou L. (2013). MicroRNA-126 regulates EPCs function: implications for a role of miR-126 in preeclampsia. *J. Cell. Biochem.* 114 2148–2159. 10.1002/jcb.24563 23553946

[B150] Yang S. Li H. Ge Q. Guo L. Chen F. (2015). Deregulated microRNA species in the plasma and placenta of patients with preeclampsia. *Mol. Med. Rep.* 12 527–534. 10.3892/mmr.2015.3414 25738738

[B151] Yang W. Wang A. Zhao C. Li Q. Pan Z. Han X. (2016). miR-125b enhances IL-8 production in early-onset severe preeclampsia by targeting sphingosine-1-phosphate lyase 1. *PLoS One* 11:e166940. 10.1371/journal.pone.0166940 27935985 PMC5147846

[B152] Yang X. Guo F. (2019). miR3423p suppresses cell migration and invasion in preeclampsia by targeting plateletderived growth factor receptor alpha. *Mol. Med. Rep.* 20 1772–1780. 10.3892/mmr.2019.10372 31257526 PMC6625458

[B153] Yang X. Guo L. Li H. Chen X. Tong X. (2012). Analysis of the original causes of placental oxidative stress in normal pregnancy and pre-eclampsia: a hypothesis. *J. Matern. Fetal. Neonatal. Med.* 25 884–888. 10.3109/14767058.2011.601367 21740314

[B154] Yang Y. Fan X. Mao M. Song X. Wu P. Zhang Y. (2017). Extensive translation of circular RNAs driven by N(6)-methyladenosine. *Cell Res.* 27 626–641. 10.1038/cr.2017.31 28281539 PMC5520850

[B155] Yang Y. Xi L. Ma Y. Zhu X. Chen R. Luan L. (2019). The lncRNA small nucleolar RNA host gene 5 regulates trophoblast cell proliferation, invasion, and migration via modulating miR-26a-5p/N-cadherin axis. *J. Cell. Biochem.* 120 3173–3184. 10.1002/jcb.27583 30242892

[B156] Yoon J. H. Abdelmohsen K. Gorospe M. (2013). Posttranscriptional gene regulation by long noncoding RNA. *J. Mol. Biol.* 425 3723–3730. 10.1016/j.jmb.2012.11.024 23178169 PMC3594629

[B157] Yu Y. Wang L. Gao M. Guan H. (2018). Long non-coding RNA TUG1 regulates the migration and invasion of trophoblast-like cells through sponging miR-204-5p. *Clin. Exp. Pharmacol. Physiol.* 46 380–388. 10.1111/1440-1681.13058 30575983

[B158] Yuan Y. Wang X. Sun Q. Dai X. Cai Y. (2020). MicroRNA-16 is involved in the pathogenesis of pre-eclampsia via regulation of notch2. *J. Cell. Physiol.* 235 4530–4544. 10.1002/jcp.29330 31643078

[B159] Zhang L. Li H. Li M. Zhang W. Yang Z. Zhang S. (2020). LRP6 is involved in the proliferation, migration and invasion of trophoblast cells via miR346. *Int. J. Mol. Med.* 46 211–223. 10.3892/ijmm.2020.4570 32319541 PMC7255486

[B160] Zhang L. Qin D. Hao C. Shu X. Pei D. (2013). SNX16 negatively regulates the migration and tumorigenesis of MCF-7 cells. *Cell. Regen.* 2:3. 10.1186/2045-9769-2-3 25408875 PMC4230745

[B161] Zhang M. Muralimanoharan S. Wortman A. C. Mendelson C. R. (2016). Primate-specific miR-515 family members inhibit key genes in human trophoblast differentiation and are upregulated in preeclampsia. *Proc. Natl. Acad. Sci. U.S.A.* 113 E7069–E7076. 10.1073/pnas.1607849113 27791094 PMC5111716

[B162] Zhang S. S. Kan X. Q. Liu P. Yin L. Z. Li Q. Y. Xu H. Y. (2020). MiR-20b is implicated in preeclampsia progression via the regulation of myeloid cell leukemin-1. *J. Biol. Regul. Homeost. Agents* 34 1709–1717. 10.23812/20-231-A 33176419

[B163] Zhang W. M. Cao P. Xin L. Zhang Y. Liu Z. Yao N. (2019). Effect of miR-133 on apoptosis of trophoblasts in human placenta tissues via Rho/ROCK signaling pathway. *Eur. Rev. Med. Pharmacol. Sci.* 23 10600–10608. 10.26355/eurrev_201912_19755 31858525

[B164] Zhang W. Yang M. Yu L. Hu Y. Deng Y. Liu Y. (2020). Long non-coding RNA lnc-DC in dendritic cells regulates trophoblast invasion viap-STAT3-mediated TIMP/MMP expression. *Am. J. Reprod. Immunol.* 83:e13239. 10.1111/aji.13239 32215978

[B165] Zhang W. Zhou Y. Ding Y. (2017). Lnc-DC mediates the over-maturation of decidual dendritic cells and induces the increase in Th1 cells in preeclampsia. *Am. J. Reprod. Immunol.* 77:e12647. 10.1111/aji.12647 28185352

[B166] Zhang Y. G. Yang H. L. Long Y. Li W. L. (2016). Circular RNA in blood corpuscles combined with plasma protein factor for early prediction of pre-eclampsia. *BJOG* 123 2113–2118. 10.1111/1471-0528.13897 26846540

[B167] Zhang Y. Cao L. Jia J. Ye L. Wang Y. Zhou B. (2019). CircHIPK3 is decreased in preeclampsia and affects migration, invasion, proliferation, and tube formation of human trophoblast cells. *Placenta* 85 1–8. 10.1016/j.placenta.2019.07.010 31421528

[B168] Zhang Y. Fei M. Xue G. Zhou Q. Jia Y. Li L. (2012). Elevated levels of hypoxia-inducible microRNA-210 in pre-eclampsia: new insightsinto molecular mechanisms for the disease. *J. Cell Mol. Med.* 16 249–259. 10.1111/j.1582-4934.2011.01291.x 21388517 PMC3823289

[B169] Zhang Y. Qu L. Ni H. Wang Y. Li L. Yang X. (2020). Expression and function of lncRNA MALAT1 in gestational diabetes mellitus. *Adv. Clin. Exp. Med.* 29 903–910. 10.17219/acem/121524 32783409

[B170] Zhang Y. Zhang X. O. Chen T. Xiang J. F. Yin Q. F. Xing Y. H. (2013). Circular intronic long noncoding RNAs. *Mol. Cell.* 51 792–806. 10.1016/j.molcel.2013.08.017 24035497

[B171] Zhang Y. Zou Y. Wang W. Zuo Q. Jiang Z. Sun M. (2015). Down-regulated long non-coding RNA MEG3 and its effect on promoting apoptosis and suppressing migration of trophoblast cells. *J. Cell Biochem.* 116 542–550. 10.1002/jcb.25004 25358633

[B172] Zhang Z. Yang T. Xiao J. (2018). Circular RNAs: promising biomarkers for human diseases. *Ebiomedicine* 34 267–274. 10.1016/j.ebiom.2018.07.036 30078734 PMC6116471

[B173] Zhao G. Miao H. Li X. Chen S. Hu Y. Wang Z. (2016). TGF-beta3-induced miR-494 inhibits macrophage polarization via suppressing PGE2 secretion in mesenchymal stem cells. *FEBS Lett.* 590 1602–1613. 10.1002/1873-3468.12200 27149081

[B174] Zhao Y. H. Liu Y. L. Fei K. L. Li P. (2020). Long non-coding RNA HOTAIR modulates the progression of preeclampsia through inhibiting miR-106 in an EZH2-dependent manner. *Life Sci.* 253:117668. 10.1016/j.lfs.2020.117668 32320706

[B175] Zhao Z. M. Jiang J. (2018). Lowly expressed EGFR-AS1 promotes the progression of preeclampsia by inhibiting the EGFR-JAK/STAT signaling pathway. *Eur. Rev. Med. Pharmacol. Sci.* 22 6190–6197. 10.26355/eurrev_201810_16024 30338781

[B176] Zhou W. Wang H. Wu X. Long W. Zheng F. Kong J. (2018). The profile analysis of circular RNAs in human placenta of preeclampsia. *Exp. Biol. Med. (Maywood)* 243 1109–1117. 10.1177/1535370218813525 30458645 PMC6327373

[B177] Zhu H. Niu X. Li Q. Zhao Y. Chen X. Sun H. (2020). Circ_0085296 suppresses trophoblast cell proliferation, invasion, and migration via modulating miR-144/E-cadherin axis. *Placenta* 97 18–25. 10.1016/j.placenta.2020.06.002 32792057

[B178] Zhu H. Zhou X. Chang H. Li H. Liu F. Ma C. (2015). CCAT1 promotes hepatocellular carcinoma cell proliferation and invasion. *Int. J. Clin. Exp. Pathol.* 8 5427–5434. 26191246 PMC4503117

[B179] Zhu X. M. Han T. Sargent I. L. Yin G. W. Yao Y. Q. (2009). Differential expression profile of microRNAs in human placentas from preeclamptic pregnancies vs normal pregnancies. *Am. J. Obstet. Gynecol.* 200:661. 10.1016/j.ajog.2008.12.045 19285651

[B180] Zou Y. Jiang Z. Yu X. Sun M. Zhang Y. Zuo Q. (2013). Upregulation of long noncoding RNA SPRY4-IT1 modulates proliferation, migration, apoptosis, and network formation in trophoblast cells HTR-8SV/neo. *PLoS One* 8:e79598. 10.1371/journal.pone.0079598 24223182 PMC3819274

[B181] Zuo Q. Huang S. Zou Y. Xu Y. Jiang Z. Zou S. (2016). The Lnc RNA SPRY4-IT1 modulates trophoblast cell invasion and migration by affecting the epithelial-mesenchymal transition. *Sci. Rep.* 6:37183. 10.1038/srep37183 27853262 PMC5112580

